# A nomenclator for the frailejones (Espeletiinae Cuatrec., Asteraceae)

**DOI:** 10.3897/phytokeys.16.3186

**Published:** 2012-08-21

**Authors:** Mauricio Diazgranados

**Affiliations:** 1 Department of Biology, Saint Louis University, 3507 Laclede Ave., St. Louis, MO 63103; 2 Missouri Botanical Garden, 4344 Shaw Boulevard, Saint Louis, MO 63110

**Keywords:** Andean forest, *Carramboa*, *Coespeletia*, *Espeletia*, *Espeletiopsis*, frailejón, *Libanothamnus*, Páramo, *Paramiflos*, *Ruilopezia*, *Tamania*

## Abstract

The páramos and high Andean forests of the tropical Andes are largely dominated by frailejones (*Nomen nudum* Cuatrec., *Nomen nudum*). These plants are ecologically and culturally essential for both ecosystems and local inhabitants. The frailejones have been studied for over two centuries, but the taxonomic knowledge is still sparse and incomplete. The inedited monograph by Cuatrecasas contains only ca. 70% of the species known today, and publications in the last decade disagree regarding the number of taxa within the group, with estimates ranging from 3 genera and 90 species to 8 genera and 154 species. Moreover the literature contains inexact information about their distribution. As part of a study of the phylogenetic and biogeographic relationships of the group, a thorough revision of the nomenclature was needed as a first step. Currently the subtribe has 8 recognized genera, 141 species, 17 subspecies, 22 varieties, 8 forms, 33 recognized hybrids, 142 synonyms and 5 invalid names, for a total of 368 names (autonyms not counted). The most current list of taxa is presented here, along with some notes and Spanish names. *Tamananthus crinitus* V.M.Badillo is not included within the subtribe. Various previous species or infraspecific taxa (i.e. *Carramboa tachirensis* (Aristeg.) Cuatrec., *Espeletia algodonosa* Aristeg., *Espeletia aurantia* Aristeg., *Espeletia brassicoidea* var.* macroclada*, *Espeletia brassicoidea* var.* pedunculata***,** *Espeletia garcibarrigae* Cuatrec. and *Espeletiopsis cristalinensis* (Cuatrec.) Cuatrec.) are proposed or confirmed as hybrids. Two new records for Colombia are mentioned: *Ruilopezia cardonae* (Cuatrec.) Cuatrec., which is the first report of *Ruilopezia* for that country, and *Espeletia steyermarkii* Cuatrec. Observations regarding the frequency of hybrids in the subtribe are also given.

## Introduction

José Celestino Mutis, founder and director of the “Expedición Botánica del Nuevo Reino de Granada” wrote two short diagnoses for the first species of frailejones to be collected, described and illustrated; this material is currently preserved at the Royal Botanical Garden of Madrid, Spain. A manuscript with a four-page description, dated March 7 1792, was found in the Institute of France, Paris. This document was presumably written by Juan Bautista Aguilar, calligrapher for the Mutis expedition, and included detailed morphological information and uses. These earliest works are evidence of the admiration that these plants caused in the members of the botanical expedition. Among all the plants collected in over a decade in the New Kingdom of Granada, Mutis chose these plants to honor the viceroy José de Ezpeleta, dedicating the genus *Espeletia* to him. The genus was published officially by Humboldt and Bonpland in Plantae *Nomen nudum* (1809 [1808]) de Cumana et de Barcelone, aux Andes de la Nouvelle Grenade, de Quito et du Pérou, et sur les bords du rio-Negro de Orénoque et de la rivière des Amazones. This was the beginning of two centuries of prolific but complex scientific research on this group.

Today new species are still being discovered and the taxonomy of some genera is still unfinished. Dr José Cuatrecasas (1903–1996), a prominent Spanish botanist, spent nearly 60 years studying the group, and published in 1976 the subtribe *Nomen nudum* Cuatrec. with seven genera: *Carramboa* Cuatrec., *Coespeletia* Cuatrec., *Espeletia* Mutis ex Humb. & Bonpl., *Espeletiopsis* Cuatrec., *Libanothamnus* Ernst, *Ruilopezia* Cuatrec. and *Tamania* Cuatrec. (Cuatrecasas 1976). The eighth genus, *Paramiflos* Cuatrec., was added almost 20 years later (Cuatrecasas 1995). The subtribe has numerous synapomorphies, such as spiral leaf phyllotaxis; obpyramidal to prismatic shape of the epappose cypselae; fertile female ray flowers and functionally male disc flowers; pluriseriate involucre and persistent pales of the receptacle; thick and woody stems; xeromorphic structure; specialized life-forms; and a static chromosome number (n=19) (Cuatrecasas 1976; Cuatrecasas in press).

The frailejones are very abundant and dominant in the páramos, subpáramos and high Andean forests (Cuatrecasas 1986). They are of critical ecological importance because they contribute to regulating the hydrologic cycle, produce most of the biomass in these ecosystems, prevent soil erosion, and have key associations with more than 125 animal species (García et al. 2005). Much like the astonishing dendrosenecios of the African mountains, frailejones have intrigued naturalists and botanists, not just for their appealing beauty but also for their remarkable adaptations to the extremely harsh environmental conditions of the páramo. Their morphological diversity is impressive. Plant sizes range from diminutive fist-sized species to plants more than 15 m high. Leaves vary from grass-like and glabrous to some of the most densely pubescent found in flowering plants. Reproductive structures exhibit a similarly spectacular level of variation and hybridization occurs between almost all sympatric species.

In the last decades hundreds of works have been produced on the páramo ecosystem, mainly on ecology, evolution, ethnobotany, and phytochemistry. A quick search in Google Scholar (accessed in March 28 2012) yields 1290 biological papers mentioning one or more of the genera of the subtribe, and 104 papers include these names in their titles. However, the lack of clarity regarding the correct nomenclature and taxonomy of the *Nomen nudum*, the obligate point of reference in almost all studies on páramos, is a serious handicap. Cuatrecasas worked until his last day on the revision of the subtribe. This revision is based on his extremely detailed morphological observations and extensive field experience of decades, and treats seven of the eight genera (excluding *Espeletiopsis*) and 105 species. The revision includes new taxa and combinations, and some of these unpublished names have been used in the literature for more than a decade. The manuscript, however, is still in press at the New York Botanical Garden, and valid publication of these names is forthcoming.

In 1996 Cuatrecasas published a key for 20 species of *Espeletiopsis*, knowing that he would not be able to finish the treatment for this genus (Cuatrecasas 1996). Since then, nearly 19 new species and one variety have been published, one species was reestablished and one hybrid proposed (Díaz-Piedrahita and Obando 2004; Díaz-Piedrahita and Pedraza 2001; Díaz-Piedrahita and Rodríguez 2008; Díaz-Piedrahita et al. 2006; Díaz-Piedrahita and Rodriguez-Cabeza 2010; Morillo and Briceño 2007); the author is currently working on the description of 17 additional new species.

Furthermore, there is no agreement regarding Cuatrecasas’ classification, and many of the genera were not resolved as monophyletic in earlier studies. Even the number of published species is unclear in recent publications. Based on molecular evidence (nrITS), Rauscher (2002) concluded that the subtribe is monophyletic and that the closest relatives are *Rumfordia* DC., *Ichthyothere* Mart. and *Smallanthus* Mack.; he proposed to replace the subtribe *Nomen nudum* by the “*Espeletia* complex” and mentioned that the group has approximately 100 species. Díaz-Piedrahita et al. (2006) indicated a total of 154 species for *Nomen nudum*. Fagua and González (2007) mentioned ca. 100 species. Panero (2007) described the subtribe with only three genera (*Carramboa*, *Espeletia* and *Tamananthus*) and 90 species, and Baldwin (2009) reported 90 species as well. The Plant List (Missouri Botanical Garden and Royal Botanic Gardens in Kew 2010), however, shows in total 145 species (*Carramboa* 7 spp., *Coespeletia* 6 spp., *Espeletia* 69 spp., *Espeletiopsis* 24 spp., *Libanothamnus* 14 spp., *Ruilopezia* 24 spp. and *Tamania* 1 sp.), and mentions 4 hybrids, 88 synonyms, 36 scientific plant names of infraspecific rank, and 24 unassessed names. The subtribe has been recently circumscribed within the tribe Millerieae Lindl, as part of the Heliantheae Alliance (Baldwin 2009; Panero 2007).

## Methods

The taxonomy of the subtribe was carefully examined. A total of 4408 specimens from personal collections and different herbaria (A, AAU, ANDES, CAS, COL, CUVC, DS, ECON, F, FMB, G, GB, GH, H, HECASA, HUA, K, M, MA, MEDEL, MER, MERF, MIN, MO, MY, MYF, NEU, NY, P, PSO, QCA, QCNE, S, U, UC, US, VALLE, VEN and WAG) were studied. A database was built and 3408 totally identified specimens were georeferenced. Plants from 1685 photographs taken during fieldwork by the author were identified and georeferenced as well, for a total of 5093 mapped individuals. Maps were generated in ArcGIS10 (ESRI) and DIVA-GIS 7.5 (Hijmans et al. 2012).

A list of all valid names including infraspecific taxa is provided here, with synonyms and basionyms. Autonyms are not included here; these are automatically established with the first instance of valid publication of a name of an infraspecific taxon under a legitimate species, in conformity with the Art. 26.3 of the ICBN (McNeill et al. 2006). To preclude valid publication in this paper of the new taxa and combinations from Cuatrecasas’ manuscript, no type information, description, diagnosis or full and direct references were added for these names (in corformity with Art. 33.4 and 33.8 of the ICBN, in McNeill et. al. 2006). In addition, the abbreviation “*ined*.” was added after the names, as well as a note about the forthcoming publication. The information for these names will be updated in the online version after after the publication of the Cuatrecasas manuscript.

Hybrids are listed at the end of each genus. The dominant morphology of the hybrid corresponds to the first species listed in the hybrid formula. Therefore hybrids are arranged alphabetically by the first species in the formula.

There is no English name for these plants. In Spanish they are usually called frailejones (pronounced fry-lay-ho-ness; in singular frailejón, pronounced fry-lay-hón), which means “big monk”, due to the resemblance of these plants to medieval monks when seen in the typically foggy weather of the páramo. This name is applied to all the species of the group. Some of the smallest species are also called chijí. The arboreal species often receive equivocal (applied to more than one species) names like anime, carrambo and quiñon (*Carramboa* spp.), punta de lanza, tabaco and its derived names, trementino or incienso (*Libanothamnus* spp. and *Tamania chardonii*). Punta de lanza refers to the spear-like shape of the leaves. Tabaco and derived homonymy (tabaquero, tabaquillo, tabacote, tabacón, etc.) were probably given by the resemblance of the leaves of some species to tobacco leaves. The names trementino and incienso originated from a common use for these plants. Resin from all the frailejones is abundant and aromatic, and can be used as incense or to extract oil of turpentine, which is used as a solvent for paints and varnishes. This was a common practice in the eighteenth century, as reported in the first description of the genus *Espeletia* by Mutis’ expedition in 1792.

There are other common names used, mainly in Venezuela, where the group shows its maximum morphological variation. A list of all common names found on specimens and in the literature (Bernal et al. 2012) contained a total of 49 different names used for all 141 species and some hybrids. There are 16 univocal names (applied only to a single species), but there are no biunivocal names (applied only to a single species, which does not receive any other common name). One species receives six common names and four species are referred to by five names.

The red list for the frailejones of Colombia (García et al. 2005) proposed new Spanish names for all the species included in the publication, with the aim of socializing the knowledge of these species and instilling a feeling of responsibility among local inhabitants to protect these resources. This strategy has been applied successfully in the past, and remarkable examples exist today, especially with birds, where some endangered species that are well-known today were virtually unknown to the public two decades ago. *Nomen nudum* are common motifs in posters, postcards and T-shirts, and most Colombians, northern Ecuadorians and Venezuelans from the mountain regions have seen or heard of frailejones. However, their knowledge about the diversity of this group is extremely low, and most people believe that there is only one species, the frailejón.

In this work, all common names reported for each taxon are included, and the respective countries of origin are indicated in parenthesis (C: Colombia, E: Ecuador, V: Venezuela). When names are in indigenous tongue, the name of the tongue is reported (e.g. Kamsá). Names for intraspecific taxa are reported only when they differ from the names used for the species. For species that lack univocal names, new univocal Spanish names are proposed to create the consciousness, appropriateness and responsibility for conservation among locals (marked with *). Recommended names to use (univocal names) are emphasized in bold.

## Systematics

**1 *Carramboa*** **Cuatrec.**, Phytologia 35(1): 54. 1976. **TYPE:** *Espeletia pittieri* Cuatrec. (= *Carramboa badilloi* var. *pittieri* (Cuatrec.) Cuatrec.)

**1.1 *Carramboa badilloi*** **(Cuatrec.) Cuatrec.**, Phytologia 35(1): 54. 1976. *Espeletia badilloi* Cuatrec., Ciencia (Mexico) 6(7–9): 261. 1945. **TYPE. VENEZUELA: Mérida:** Páramo de Don Pedro, 2900 m, small tree, yellow ligules, 18 Jul 1944, V.M.Badillo 991 (holotype: VEN).

*Carramboa littlei* (Aristeg.) Cuatrec., Phytologia 35(1): 54. 1976. *Espeletia littlei* Aristeg., Fl. Venez. 10(1): 433–435, fig. 67, p. 434. 1964. **TYPE. VENEZUELA: Mérida:** La Carbonera, 2700 m, Oct 1953, E.L.Little 15592 (holotype: VEN; isotype: VEN).

**Common names.** **Anime amarillo** (V), anime montañero (V), carrambo (V), carrambo paramero (V), frailejón amarillo (V), frailejón anime (V).

**Note.** *Carramboa badilloi* includes now former *Carramboa littlei* and *Carramboa pittieri* was proposed as a variety of *Carramboa badilloi* (below). However some individuals show contrasting morphological differences, especially in the morphology of the leaves. More ecological, morphological, anatomical and genetic studies are needed to determine if these entities are indeed different taxa.

***Carramboa badilloi* var. *pittier*i** **(Cuatrec.) Cuatrec.**, *ined*. (Forthcoming publication in Cuatrecasas in press.)

*Carramboa pittieri* (Cuatrec.) Cuatrec.

*Espeletia pittieri* Cuatrec.

**Common names.** Carrambo (V), anime amarillo (V), **anime montañero** (V).

**1.2 *Carramboa rodriguezii*** **(Cuatrec.) Cuatrec.**, Phytologia 35(1): 54. 1976. *Espeletia rodriguezii* Cuatrec., Phytologia 29(5): 379–382. 1975. **TYPE. VENEZUELA: Mérida:** Betania, between Páramo de Las Coloradas and El Molino, 2400 m, Jun 1974, M.López-Figueiras & H.Rodríguez 9050 (holotype: US!; isotypes: F!, MERF!).

**Common names.** Carrambo (V), carrambo de las Coloradas*.

**Note.** No large populations of this species have been observed and it might be an intergeneric hybrid.

**1.3 *Carramboa trujillensis*** **(Cuatrec.) Cuatrec.**, Phytologia 35(1): 54. 1976. *Espeletia trujillensis* Cuatrec., Mutisia 16: 5–7, figs. 4, 5T. 1953. **TYPE. VENEZUELA: Trujillo:** Quebrada del Cortijo, above Humocaro Bajo, 2600–2800 m, 6 Feb 1944, J.Steyermark 55341 (holotype: F!; isotype: F!).

**Common names.** Anime (V), carrambo (V), **quiñón** (V).

**1.4 *Carramboa wurdackii*** **(Ruiz-Terán & López-Fig.) Cuatrec.**, Phytologia 52(3): 158. 1982. *Espeletia wurdackii* Ruiz-Terán & López-Fig., Rev. Fac. Farm. Univ. Andes Mérida 17: 1–5, figs. 1–2. 1976. *Libanothamnus wurdackii* (Ruiz-Terán & López-Fig.) Cuatrec., Phytologia 35(1): 51. 1976. **TYPE. VENEZUELA: Trujillo:** Finca Florencia, 2450 m, near Carache, 3 Apr 1976, M.López-Figueiras 12957 (holotype: MERF!; isotype: NY!, US!).

**Common names.** Carrambo (V), carrambo de Carache*.

**Note.** This species is known from only a few collections; it may be an intergeneric hybrid.

**Hybrids**

***Carramboa ×tachirensis*** **(Aristeg.) Cuatrec. (= *Carramboa badilloi* var. pittieri (Cuatrec.) Cuatrec.** × ***Ruilopezia marcescens* (S. F. Blake) Cuatrec.)**, Phytologia 52(3): 158. 1982 (hybrid status in Rev. Fac. Agron. 1:475–481. 2007). *Espeletia tachirensis* Aristeg., Fl. Venez. 10(1): 427–428. 1964. **TYPE. VENEZUELA: Táchira:** Páramo del Batallón, 26 Sep 1956, L.Aristeguieta 2523 (holotype: VEN; isotype: US!).

*Espeletiopsis tachirensis* (Aristeg.) Cuatrec., Phytologia 35(1): 56. 1976.

**Common names.** Carrambo (V), carrambo de Táchira*.

**Note.** Molecular analyses of ITS and ETS regions by the author confirm the hybrid nature of this combination (Diazgranados 2012). Anatomical and morphological studies support this conclusion (Morillo and Briceño 2007).

**2 *Coespeletia*** **Cuatrec.**, Phytologia 35(1): 55–56, fig. 4, pl. 61. 1976. **TYPE.** *Coespeletia spicata* (Sch. Bip. ex Wedd.) Cuatrec.

**2.1 *Coespeletia albarregensis*** **Cuatrec.**, *ined*. (Forthcoming publication in Cuatrecasas in press.)

**Common names.** **Albarraguero** (V), frailejón (V)**.**

**2.2 *Coespeletia elongata*** **(A. C. Sm.) Cuatrec.**, Phytologia 35(1): 57. 1976. *Espeletia elongata* A. C. Sm., Amer. J. Bot. 27: 546. 1940. **TYPE. VENEZUELA: Mérida:** Páramo de los Conejos, 4000 m, 10 Sep 1983, J.Hanbury-Tracy 83 (holotype: NY!; isotype: K).

**Common names.** Frailejón (V), frailejón amarillo (V), frailejón florón*.

**2.3 *Coespeletia laxiflora*** **(S. Díaz & Rodr.-Cabeza) S. Díaz & Rodr.-Cabeza**, Revista Acad. Colomb. Ci. Exact. 35(137): 422, 424. 2011. *Espeletiopsis laxiflora* S. Díaz & Rodr.-Cabeza, Revista Acad. Colomb. Ci. Exact. 34 (133): 441–454, figs. 1, 3A–B. 2010. **TYPE. COLOMBIA: Santander:** Santuario de Fauna y Flora Guanentá Alto Río Fonce, Municipio de Encino, Vda. Avendaños Tres, sector Páramo de Las Playas, 19 Oct. 2008, B. V. Rodríguez-Cabeza 1993 (holotype: COL; isotypes: COL!, HUA, UIS, UPTC).

**Common names.** Frailejón (C), frailejón laxo*.

**2.4 *Coespeletia moritziana*** **(Sch. Bip. ex Wedd.) Cuatrec.**, Phytologia 35(1): 57. 1976. *Espeletia moritziana* Sch. Bip. ex Wedd., Chlor. And. 1: 65–66. 1856. **TYPE. VENEZUELA: Mérida:** Sierra Nevada, 4000–4500 m, 1842, J.J.Linden 399 (lectotype: P; J.J.Linden 368 and 398 isolectotypes: P, US!).

*Espeletia rufescens* Cuatrec., Bol. Soc. Venez. Ci. Nat. 17 (85): 88–91, figs. 5, 6. 1956. **TYPE. VENEZUELA: Mérida:** Sierra Nevada de Mérida, 12000 ft, J.J.Linden 398, (lecto-holotype: P, only leaves; J.J.Linden 399 paratype: P, only leaves pro parte).

**Common names.** Frailejón (V), frailejón amarillo (V), **frailejón cabello de ángel** (V), frailejón dorado (V), frailejón morado (V).

**Note.** *Espeletia rufescens* is an herbarium mixture and only the leaves of the type collection of *Espeletia rufescens* correspond to *Coespeletia moritziana* (see *Espeletia rufescens* below).

**2.5 *Coespeletia spicata*** **(Sch. Bip. ex Wedd.) Cuatrec.**, Phytologia 35(1): 57. 1976. *Espeletia spicata* Sch. Bip. ex Wedd., Chlor. And. 1: 65. 1856. **TYPE. VENEZUELA: Mérida:** Sierra Nevada de Mérida, 14000 ft, Aug 1842, J.J.Linden 400 (lectotype: P; isolectotypes K!, FI).

*Coespeletia alba* (A. C. Sm.) Cuatrec., Phytologia 35(1): 57. 1976. *Espeletia alba* A. C. Sm., Brittonia 1(7): 512–513. 1935. **TYPE. VENEZUELA: Mérida:** Sierra Nevada de Mérida, Cordillera del Norte, Páramo de Mucurubá El Rincon, El Colorado Fila de Estiti, 3900 m, Apr 1930, W.Gehriger 125 (holotype: NY!; isotypes: F!, G!, MO!, US!, VEN, photo holotype 4789: F).

**Common names.** Frailejón (V), frailejón amarillo (V), frailejón blanco (V), frailejón gris (V), frailejón morado (V), frailejón pincho*.

**2.6 *Coespeletia thyrsiformis*** **(A. C. Sm.) Cuatrec.**, Phytologia 35(1): 57. 1976. *Espeletia thyrsiformis* A. C. Sm., Brittonia 1(7): 513. 1935. **TYPE. VENEZUELA: Táchira:** wrongly cited from “Páramo de Mucuchíes”, Dec 1927, R.Gutzviller 36 (holotype: US!; isotypes: G!, NY!).

**Common names.** Frailejón (V), frailejón amarillo del batallón*.

***Coespeletia thyrsiformis* f. *marcan*a** **(Cuatrec.) Cuatrec.**, *ined*. (Forthcoming publication in Cuatrecasas in press.)

*Coespeletia marcana* (Cuatrec.) Cuatrec.

*Espeletia marcana* Cuatrec.

*Espeletia racemosa* Cuatrec.

**2.7 *Coespeletia timotensis*** **(Cuatrec.) Cuatrec.**, Phytologia 35(1): 57. 1976. *Espeletia timotensis* Cuatrec., Bol. Soc. Venez. Ci. Nat. 17(85): 84, figs. 1, 2. 1956. **TYPE. VENEZUELA: Mérida:** Sierra Nevada, Páramo de Timotes–Piñango, 4000 m, open páramo, 9 Dec 1938, J.Hanbury-Tracy 193 (holotype: K; isotypes: NY!, US!).

*Coespeletia lutescens* (Cuatrec. & Aristeg.) Cuatrec., Phytologia 35(1): 57. 1976. *Espeletia lutescens* Cuatrec. & Aristeg., Fl. Venez. 10(1): 443–444. 1964. **TYPE. VENEZUELA: Mérida:** Páramo de Timotes, 3000–3500 m, 4000 m on tag, Dec 1910, A. Jahn 149 (holotype: US!; isotypes: G, VEN).

**Common names.** Frailejón (V), frailejón amarillo (V), frailejón blanco (V), **frailejón bravo** (V), frailejón morado (V).

**Hybrids**

***Espeletia ×aurantia* Aristeg. (= *Coespeletia moritziana* (Sch. Bip. ex Wedd.) Cuatrec.** × ***Espeletia schultzii* Wedd.)**, Fl. Venez. 10(1): 448. 1964. **TYPE. VENEZUELA: Mérida:** Laguna Verde, 4100 m, Oct 1956, L.Aristeguieta 2613 (holotype: VEN).

**Note.** Only a few individuals of this hybrid combination can be seen in the topolocality in the surroundings of Laguna Verde, where *Coespeletia moritziana* and *Espeletia schultzii* have abundant populations. Morphological characters described for *Espeletia aurantia* are not consistent and are certainly a mix between the parental species proposed here. The large capitula with carnose and rather short orange ray flowers is typical for *Coespeletia moritziana*. The leaves, which are broader than in *Coespeletia moritziana*, the large opposite branchlets of the synflorescence and the hirsute ray corollas suggest introgression from *Espeletia schultzii*.

***Coespeletia moritziana* (Sch. Bip. ex Wedd.) Cuatrec. *× C. timotensis* (Cuatrec.) Cuatrec.** **Representative specimens. VENEZUELA: Mérida:** alrededores del Picacho de El Águila, 12 Nov 1952, L.Aristeguieta 1076 (US!); ibidem: Sierra Nevada, páramos alrededores de Picos Bolívar y Espejo, 4600 m, 15 Dec 1959, H.G.Barclay 10187 (US!); ibidem: Páramo de Las Cruces (Páramo de Mucuchíes), a 6,3 km de El Águila por la carretera a Piñango, Distritos Rangel y Justo Briceño, 4220 m, caulirrosula de 1/2 m de altura. sinflorescencias axilares, racemosas, capítulos con lígulas rojizas, hojas amarillas en la base, 11 Jun 1983, P.E.Berry 4293 (US!, MO!); ibidem: Páramo de Mucuchíes, el Águila, 4200 m, 5 Oct 1969, J.Cuatrecasas 28029 (US!); ibidem: Páramo de Mucuchíes, cabeceras del río Chama, cercanías del Pico El Águila, Cordillera de los Andes, 3900–4000 m, 5 Oct 1977, M.López-Figueiras 14023 (US!).

***Coespeletia spicata* (Sch. Bip. ex Wedd.) Cuatrec. *× C. timotensis* (Cuatrec.) Cuatrec.** **Representative specimens.** **VENEZUELA: Mérida:** Miranda, páramo en las cabeceras de la Quebrada El Turmero (afluente del Río Motatán), a 11.7 km de El Águila por la carretera a Piñango, 4160 m, 12 Nov 1984, P.E.Berry 4399 (US!); ibidem: a 10.8 km de El Águila por la carretera a Piñango, 4180 m, 12 Nov 1984, P.E.Berry 4400 (US!); ibidem: a 11.5 km de El Águila por la carretera a Piñango, 4180 m, 12 Nov 1984, P.E.Berry 4401 (US!).

***Coespeletia timotensis* (Cuatrec.) Cuatrec.** × ***Espeletia schultzii* Wedd.** **Representative specimens. VENEZUELA: Mérida:** Páramo de Las Cruces (Páramo de Mucuchíes), a 6,3 km de El Águila por la carretera a Piñango, Distritos Rangel y Justo Briceño, 4300 m, 17 Dec 1982, P.E.Berry 3996(a) (US!).

**3 *Espeletia*** **Mutis ex Humb. & Bonpl.,** Pl. Aequinoc. 2: 10. 1808. **TYPE.** *Espeletia grandiflora* Humb. & Bonpl.

**3.1 *Espeletia annemariana*** **Cuatrec.**, Phytologia 32(4): 315–317. 1975. **TYPE. COLOMBIA: Boyacá:** Alto de Mogotes, from Vadohondo to Labranzagrande, subpáramo, 3300 m, 2 Apr 1973, A.M.Cleef 9296 (holotype: US!; isotypes: COL!, U).

**Common names.** Frailejón (C), frailejón blanco*.

***Espeletia annemariana* var. *rupicol*a** **Cuatrec.**, Phytologia 32(4): 317. 1975. **TYPE. COLOMBIA: Boyacá:** Peña del Arnical headwaters of Quebrada Candelas, rocky cliffs, 3600 m, 6 Apr 1973, A.M.Cleef 9466 (holotype, US!; isotypes: COL!, U).

**Common names.** Frailejón (C), frailejón blanco de las rocas*.

**3.2 *Espeletia arbelaezii*** **Cuatrec.**, Revista Acad. Colomb. Ci. Exact. 3(11): 247–248, figs. 2, 6E, pl. B. 1940. **TYPE. COLOMBIA: Boyacá:** Páramo de Güina (o de Huínas), 3300 m, 19 Sep 1938, J.Cuatrecasas & H.García-Barriga 1964 (holotype: COL!; isotypes: COL!, F!, US!).

**Common names.** Frailejón (C), frailejón de Pérez-Arbeláez (C), frailejón orejudo*.

**Note.** This is a polymorphic species that needs to be revised.

**3.3 *Espeletia argentea*** **Humb. & Bonpl.**, Pl. Aequinoc. 2: 14–15, pl.7, Nov. 1808. **TYPE. COLOMBIA: Cundinamarca:** Zipaquirá, reg. Novae Granatae, ano 1801, Humboldt & Bonpland (holotype: P, herb Bonpland; fragment in F).

*Espeletia nivea* Moritz ex Wedd., nom. illeg. Chlor. And. 1: 65. 1856 [1855 publ. 30 Jun 1856].

**Common names.** Frailejón de hoja lisa (C), Frailejón plateado (C), frailejón plateado de Cundinamarca*.

***Espeletia argentea* f. *phaneracti*s** **(S. F. Blake) Cuatrec.**, Phytologia 27(3): 179. 1973. *Espeletia argentea* subsp. *phaneractis* S. F. Blake, Contr. U.S. Natl. Herb. 22(8): 603-604. 1924. *Espeletia phaneractis* (S. F. Blake) A. C. Sm., Brittonia 1(7): 525–526, Pl. 2, figs. 49–50. 1935. **TYPE. COLOMBIA: Cundinamarca:** Zipaquirá, dry Páramo on Mt. Águila West of city, 3100–3300 m, 20–24 Oct 1917, f.W.Pennell 2522 (holotype: US!; isotypes: F!, GH, MO!, NY!).

**Common names.** Frailejón de hoja lisa (C), frailejón plateado (C), frailejón plateado de Cundinamarca*.

**3.4 *Espeletia ariana*** **S. Díaz & Rodr.-Cabeza**, Revista Acad. Colomb. Ci. Exact. 30 (16): 343–345 figs. 7, 8C–D. 2006. **TYPE. COLOMBIA: Boyacá:** Municipio de Socotá, Vereda Comeza Hoyada, sector Pantano Hondo, lado izquierdo arriba de la quebrada Pantano Hondo, 3600 m alt., 19 oct 2005. B. V. Rodríguez-Cabeza, L. Velasco & E. Benítez 1454 (holotype: COL; isotype: UIS).

**Common names.** Frailejón (C), frailejón del caminante*.

**3.5 *Espeletia aristeguietana*** **Cuatrec.**, Phytologia 27(3): 174–176. 1973. **TYPE. VENEZUELA: Trujillo:** Páramo La Cristalina, La Cañada 2500–2600 m, J.Cuatrecasas, L.Ruiz-Terán & M.López-Figueiras 28194 (holotype: US!).

**Common names.** Frailejón (V), frailejón de la Cristalina*.

**3.6 *Espeletia azucarina*** **Cuatrec.**, Phytologia 47(1): 12–13. 1980. **TYPE. COLOMBIA: Boyacá:** Cerro Pan de Azúcar, north of Belén, 4000 m, A.M.Cleef 9835 (holotype: US!; isotypes: COL!, HPUJ!, U!, US!).

**Common names.** Frailejón (C), **frailejón de Pan de Azúcar** (C).

**3.7 *Espeletia barclayana*** **Cuatrec.**, Phytologia 38(1): 8–12. 1977. **TYPE. COLOMBIA: Cundinamarca:** Represa del Neusa, hill at NW, 3650 m, 26 May 1972, A.M.Cleef & Jaramillo 4174 (holotype: US!; isotypes: COL!, U).

**Common names.** Frailejón (C), frailejón repollo de Cundinamarca*.

**3.8 *Espeletia batata*** **Cuatrec.**, Phytologia 40(1): 27–29. 1978. **TYPE. VENEZUELA: Mérida:** Páramo de Los Granates, Alto del Morato, 3600 m, 10 Oct 1969, J.Cuatrecasas, L.Ruiz-Terán, M.López-Figueiras 28058 (holotype: US!; isotypes: MERF!, US!).

**Common names.** **Frailejón batato** (V), frailejón patata (V), pata de burro (V).

**3.9 *Espeletia boyacensis*** **Cuatrec.**, Phytologia 27(3): 176–179. 1973. **TYPE. COLOMBIA: Boyacá:** Alto de Canutos, open páramo, 3350 m, J.Cuatrecasas & Rodriguez 27759 (holotype: US!; isotype: COL).

*Espeletia phaneractis* subsp. *boyacensis* Cuatrec., Revista Acad. Colomb. Ci. Exact. 4(14): 167, figs. 9, 12J. 1941. **TYPE. COLOMBIA: Boyacá:** near Las Gaitas, 3300 m, Boyacá, COLOMBIA: J.Cuatrecasas 10364A (holotype: COL).

**Common names.** Frailejón plateado (C), frailejón plateado boyacense*.

**3.10 *Espeletia brachyaxiantha*** **S. Díaz**, Mutisia 37: 5–10, fig. 2. 1972. **TYPE. COLOMBIA: Boyacá:** Páramo Alto de Las Cruces, NW of Belén, 3800 m, 6 Mar 1972, A.M.Cleef 2326 (holotype: COL!; isotypes: U, US!).

**Common names.** Frailejón (C), **frailejón de Belén** (C).

**3.11 *Espeletia brassicoidea*** **Cuatrec.**, Revista Acad. Colomb. Ci. Exact. 4(15–16): 337–338, figs. 1, 6A, 6B, pl. I, II. 1941. **TYPE. COLOMBIA: Norte de Santander:** Páramo de Fontibón, 2700 m, 21 Jun 1940, J.Cuatrecasas & H.García-Barriga 10096 (holotype: COL!; isotypes: COL!, US!).

*Espeletia brassicoidea* f. *pamplonensis* Cuatrec., Revista Acad. Colomb. Ci. Exact. 5(17): 338. 1942.*Nomen nudum*.

**Common names.** Frailejón (C, V), **frailejón arrepollado** (C).

***Espeletia brassicoidea* subsp. *angust*a** **Cuatrec.**, Phytologia 47(1): 13. 1980. **TYPE. COLOMBIA: Norte de Santander:** Páramo between Pamplona and Berlín, 3050 m, 23 Sep 1969, J.Cuatrecasas & L.Rodríguez 27916 (holotype: US!; isotype: COL).

***Espeletia brassicoidea* subsp. *constrict*a** **(Cuatrec.) Cuatrec.**, Revista Acad. Colomb. Ci. Exact. 5(17): 23. 1942. *Espeletia brassicoidea* f. *constricta* Cuatrec., Revista Acad. Colomb. Ci. Exact. 5(17): 23. 1942. **TYPE. COLOMBIA: Norte de Santander:** Páramo de Tamá, toward La Cueva, 3100 m, 28 Oct 1941, J.Cuatrecasas, R.E.Schultes & E.Smith 12653B (holotype: COL!; isotypes: F!, US!).

*Espeletia brassicoidea* f. *minorifolia* Cuatrec., Revista Acad. Colomb. Ci. Exact. 5(17): 23, pl. II. 1942. **TYPE. COLOMBIA: Norte de Santander:** Páramo de Tamá ca. La Cueva, 3100–3200 m, 28 Oct 1941, J.Cuatrecasas, Schultes & Smith 12653 (holotype: COL; isotypes: F!, US!).

**3.12 *Espeletia cabrerensis*** **Cuatrec.**, Phytologia 32(4): 318–320. 1975. **TYPE. COLOMBIA: Cundinamarca:** Cabrera, to Páramo de Sumapaz 3200 m, 23 Feb 1970, Uribe-Uribe 6400 (holotype: US!; isotype: COL).

**Common names.** Frailejón (C), **frailejón de Cabrera** (C).

**3.13 *Espeletia cachaluensis*** **Rodr.-Cabeza & S. Díaz**, Revista Acad. Colomb. Ci. Exact. 32(125): 459–462 figs. 2d, 3. 2008. **TYPE. COLOMBIA: Santander:** Santuario de Fauna y Flora Guanentá Alto Río Fonce. Municipio de Encino Vereda Avendaños, sector Los cuadros. 3744 m alt., 12 oct 2007. B.V.Rodr.-Cabeza, H.Palacios, R.Rivero & J.Velasco 1897 (holotype: COL; isotypes: COL!, UIS).

**Common names.** Frailejón (C), frailejón de Cachalú*.

**3.14 *Espeletia canescens*** **A. C. Sm.**, Brittonia 1(7): 516–517. 1935. **TYPE. COLOMBIA: Boyacá:** Páramo del Romeral, sobre La Baja, 3800–4200 m alt. 30 Ene 1927. E.P.Killip & A. C. Smith 18624 (holotype: NY!; isotypes: GH, PH, US!).

**Common names.** Frailejón (C), frailejón blanco de Santurbán*.

**Note.** This species is known only from the type collection, and it might be a hybrid of *Espeletia conglomerata*. Unfortunately the páramo where the species is found is currently threatened by mining.

**3.15 *Espeletia cayetana*** **Cuatrec.**, Phytologia 52(3): 159. 1982. *Espeletia grandiflora* var. *cayetana* Cuatrec., Phytologia 32(4): 323. 1975. **TYPE. COLOMBIA: Cundinamarca:** Páramo between Cogua and San Cayetano, near Laguna Seca, east slope of Filo del Santuario, valley 2 km south of Laguna Seca, subpáramo scrub, 3650 m alt., 17 Nov 1972, A.M.Cleef 6508 (holotype, US!; isotypes: COL!, HPUJ!, NY!, U!, US!).

**Common names.** Frailejón (C), frailejón de San Cayetano (C).

**3.16 *Espeletia chocontana*** **Cuatrec.**, Revista Acad. Colomb. Ci. Exact. 4(14): 164, figs. 4, 12G, pl. III. 1941. **TYPE. COLOMBIA: Cundinamarca:** Páramo de Chocontá, 2800 m, J.Cuatrecasas 9658 (holotype: COL; isotypes: F!, US!).

**Common names.** Frailejón (C), frailejón de Chocontá (C).

**3.17 *Espeletia chontalensis*** **Rodr.-Cabeza & S. Díaz**, Revista Acad. Colomb. Ci. Exact. 32(125): 462–464 figs. 2e–f, 4. 2008. **TYPE. COLOMBIA: Santander:** Santuario de Frauna y Flora Guanentá Alto Río Fonce. Municipio de Encino, Vereda Río Negro, camino sector Chontales–Páramo de la Rusia. 28 jun. 2007. B.V.Rodr.-Cabeza & H.Palacios 1874 (holotype: COL; isotypes: COL!, UIS).

**Common names.** Frailejón (C), frailejón de Chontales*.

**3.18 *Espeletia cleefii*** **Cuatrec.**, Phytologia 32(4): 312–314. 1975. **TYPE. COLOMBIA: Boyacá:** Sierra Nevada de Cocuy, Boqueron de Cusiri, A.M. Cleef & P.A. Florschutz 5922 (holotype: US!; isotypes: COL!, U).

**Common names.** Frailejón (C), frailejón del Cocuy*.

**3.19 *Espeletia congestiflora*** **Cuatrec.**, Revista Acad. Colomb. Ci. Exact. 3(12): 434–435, figs. 19, 23J, pl. IV. 1940. **TYPE. COLOMBIA: Boyacá:** Páramo de Guantiva near Las Gaitas, 3300 m, J.Cuatrecasas 10366 (holotype: COL!; isotypes: F!, P, US!).

**Common names.** Frailejón (C), frailejón de bastón*.

**3.20 *Espeletia conglomerata*** **Cuatrec.**, Brittonia 1(7): 515–516. 1935. **TYPE. COLOMBIA: Norte de Santander:** Páramo del Romeral, 3800–4200 m, 30 Jan 1927, E.P.Killip & A.C.Smith 18635 (holotype: NY!; isotypes: F!, GH, PH, S, US!).

*Espeletia brassicoidea* f. *contracta* Cuatrec., Revista Acad. Colomb. Ci. Exact. 5(17):23–24. 1942. **TYPE. COLOMBIA: Norte de Santander:** Páramo de Tamá at La Cueva, 3200 m, 28 Oct 1941, J.Cuatrecasas, Schultes & Smith 12653D (holotype: COL; isotype: F).

**Common names.** Frailejón (C, V), frailejón aglomerado (C).

**3.21 *Espeletia cuniculorum*** **Cuatrec.**, Phytologia 40(1): 25–27. 1978. **TYPE. VENEZUELA: Mérida:** Páramo de los Conejos, Cañada de los Puentes, 3350 m, 19 Oct 1972, L.Ruiz-Terán 7722 (holotype: US!; isotypes: HPUJ!, MERF!).

**Common names.** Frailejón (V), frailejón de los conejos*.

**3.22 *Espeletia curialensis*** **Cuatrec.**, Phytologia 20(8): 473–475. 1971. **TYPE. COLOMBIA: Boyacá:** Páramo entre Chita y Sacama, vertiente oriental (tributaria del Río Casanare), J.Cuatrecasas & L.Rodriguez 27790 (holotype: US!; isotype: COL!).

**Common names.** Frailejón (C), frailejón del Curial*.

***Espeletia curialensis* var. *exigu*a** **Rodr.-Cabeza & S. Díaz**, Revista Acad. Colomb. Ci. Exact. 30 (16): 347–349 figs. 10, 12C–D. 2006. **TYPE. COLOMBIA: Casanare:** Parque Nacional Natural El Cocuy, Municipio de La Salina, sector El Ahogadero, 3200 m **alt.,** 12 dic 2005. B.V.Rodríguez-Cabeza, J.M.Valderrama & O.E.López 1537 (holotype: COL; isotypes: COL!, UIS).

**3.23 *Espeletia discoidea*** **Cuatrec.**, Revista Acad. Colomb. Ci. Exact. 3(12): 437–438, figs. 21, 22, 23M, pl. IV. 1940. **TYPE. COLOMBIA: Boyacá:** Páramo de Guantiva below Alto de Canutos, 3200 m, 3 Aug 1940, J.Cuatrecasas 10358 (holotype: COL!; isotypes: COL!, F!, P, US!).

**Common names.** Frailejón (C), frailejón discoideo (C).

***Espeletia discoidea* var. *brevi*s** **Cuatrec.**, Revista Acad. Colomb. Ci. Exact. 3(12): 437–438, figs. 21, 22, 23 M, pl. IV. 1940. **TYPE. COLOMBIA: Boyacá:** Páramos NW of Belen, headwaters of Quebrada Laguna Grande, NW slope of the dividing ridge with dry graminetum of Calamagrostis effusa, 3830 m, 6 May 1973, A.M.Cleef 9774 (holotype: US!; isotypes: COL!, U).

**Common names.** Frailejón (C), frailejón discoideo (C).

**3.24 *Espeletia dugandii*** **Cuatrec.**, Revista Acad. Colomb. Ci. Exact. 4(14): 163–164, figs. 3, 12H, pl. III. 1941. **TYPE. COLOMBIA: Santander:** Peralonso, Páramo del Almorzadero, 3200 m, 19 Jul 1940, J.Cuatrecasas & H.García-Barriga 9889 (holotype: COL!; isotypes: F!, US!).

**Common names.** Frailejón (C), frailejón de Cerrito*, frailejón de Dugand (C).

**3.25 *Espeletia episcopalis*** **S. Díaz & Rodr.-Cabeza**, Revista Acad. Colomb. Ci. Exact. 30 (16): 341–343 figs. 6, 8C–D. 2006. **TYPE. COLOMBIA: Boyacá:** Municipio de Socotá, Vereda Comeza Hoyada, sector río Arzobispo, parte alta, Los Corazones, alrededores de las lagunas Larga y Peña Negra. 3700 m **alt.,** 21 Oct. 2005. B.V.Rodr.-Cabeza, L.Velasco & E.Benitez 1498 (holotype: COL; isotype: UIS).

**Common names.** Frailejón (C), frailejón del Arzobispo*.

**3.26 *Espeletia estanislana*** **Cuatrec.**, Revista Acad. Colomb. Ci. Exact. 3(12): 429, figs. 10, 23–A, pl. I. 1940. **TYPE. COLOMBIA: Santander:** Páramo del Almorzadero, 3700–3800 m, Hno. A.Miguel s.n. (holotype, US!).

**Common names.** Frailejón (C), frailejón de Estanislao (C), frailejón unifloro*.

**3.27 *Espeletia formosa*** **S. Díaz & Rodr.-Cabeza**, Revista Acad. Colomb. Ci. Exact. 30 (16): 336–339 figs. 3, 4E–F. 2006. **TYPE. COLOMBIA: Boyacá:** Parque Nacional Natural Pisba. Municipio de Socotá, Vereda Chipa viejo, ruta libertadora, sector El Santuario. 3414m alt. 16 Jun 2005. B.V.Rodríguez-Cabeza & L.Velasco 1407 (holotype: COL; isotype: COL!).

**Common names.** Frailejón (C), frailejón elegante*.

**3.28 *Espeletia frontinoensis*** **Cuatrec.**, Phytologia 38(1): 15–17. 1977. **TYPE. COLOMBIA: Antioquia:** Cordillera Occidental, Páramo de Frontino near Llano Grande, J.D.Boeke & J.B.McElroy 234 (holotype: US!; isotype: NY!).

**Common names.** Frailejón (C), frailejón de Frontino*.

**3.29 *Espeletia grandiflora*** **Humb. & Bonpl.**, Pl. Aequinoc. 2: 11–13. 1808. 1809 (t.p.). **TYPE.** Plant Aequinoc, Santa Fe de Bogotá et Quindio, Herbier de l’Amerique equatoriale, donné par M.A.Bonpland, Humboldt & Bonpland in “Herb. HBK” (holotype: P (fiche 112); isotype: P; photo FM-15154: US, B, from destroyed isotype, no more extant).

*Espeletia grandiflora* f. *longiligulata* Cuatrec., Revista Acad. Colomb. Ci. Exact. 4(14): 169, fig. 12C. 1941. **TYPE. COLOMBIA: Cundinamarca:** Páramo de Cruz Verde–Bogotá, 3400–3500 m, J.Cuatrecasas 10467 (holotype: COL!; isotype: US!).

*Espeletia grandiflora* f. *multiflora* Cuatrec., Revista Acad. Colomb. Ci. Exact. 4(14): 169, pl. 4. 1941. **TYPE. COLOMBIA: Cundinamarca:** Páramo de Zipaquirá, 3100–3200 m, J.Cuatrecasas 9527 (holotype: COL!; isotypes: F!, US!).

*Espeletia grandiflora* f. *reducta* Cuatrec., Revista Acad. Colomb. Ci. Exact. 4(14): 169. 1941. **TYPE. COLOMBIA: Cundinamarca:** Páramo de Zipaquirá, 3100–3200 m, J.Cuatrecasas 9527A (holotype: COL!; isotype: US!).

**Common names.** Frailejón (C), frailejón mayor*.

***Espeletia grandiflora* subsp. *boyacan*a** **(Cuatrec.) Cuatrec.**, *ined*. (Forthcoming publication in Cuatrecasas in press.)

*Espeletia grandiflora* var. *boyacana* Cuatrec.

***Espeletia grandiflora* subsp. *subnivali*s** **(Cuatrec.) Cuatrec.**, *ined*. (Forthcoming publication in Cuatrecasas in press.)

*Espeletia grandiflora* var. *subnivalis* Cuatrec.

***Espeletia grandiflora* var. *attenuat*a** **Cuatrec.**, Phytologia 32(4): 325–326. 1975. **TYPE. COLOMBIA: Cundinamarca:** Lagunas de Chisacá, Macizo de Bogotá, 3650–3700 m, 29 Dec 1959, J.Cuatrecasas & R. Jaramillo 25748 (holotype: US!; isotypes: BC!, F!, COL!, HPUJ!).

**3.30 *Espeletia hartwegiana*** **Sch. Bip. ex Cuatrec.**, Trab. Mus. Nac. Ci. Nat., Ser. Bot. 26: 17, pl. I, II-2. 1933. Ibid. 27: 105–114, pl. 23–25. 1934. Ibid. 33: 140. 1936. **TYPE. COLOMBIA: Cauca:** Páramo de Guanacas, 1841–42, C.T.Hartweg 1137 (lectotype: G (photo FM-28724); isolectotypes: P, FI, G, K!, LE, W).

*Espeletia hartwegiana* Sch. Bip., in Wedd., Chlor. And. 1: 62. 1856. Pro syn. sub *Espeletia grandiflora* Humb. & Bonpl., Pl. Aequinoc. 2: 11–13. 1808. 1809 (t.p.).

*Espeletia grandiflora* var. *hartwegiana* (Cuatrec.) Benoist, Bull. Soc. Bot. France 92: 139. 1945.

**Common names.** Frailejón (C), frailejón amarillo (C), frailejón de Los Nevados*.

***Espeletia hartwegiana* subsp. *barragensi*s** **Cuatrec.**, Phytologia 45(1): 23–24. 1980. **TYPE. COLOMBIA: Valle del Cauca:** Páramo de Santa Lucía, above Barragán, 3600–3680 m, 16 Mar 1946, J.Cuatrecasas 20076 (holotype: COL!; isotypes: BC!, F!, HPUJ!, P, US!).

**Common names.** Frailejón (C), frailejón de Barragán*.

***Espeletia hartwegiana* subsp. *centroandin*a** **Cuatrec.**, Phytologia 45(1): 21–33. 1980. **TYPE. COLOMBIA: Caldas:** Nevado del Ruiz, El Aprisco, 3600–3800 m, 5 May 1940, J.Cuatrecasas 9312 (holotype: COL!; isotypes: F!, HPUJ!, US!).

*Espeletia centroandina* Cuatrec., pro syn. sub *Espeletia hartwegiana* Sch. Bip. ex Cuatrec., Trab. Mus. Nac. Ci. Nat., Ser. Bot. 26: 17. 1933.

**Common names.** Frailejón (C), frailejón de Los Nevados*.

***Espeletia hartwegiana* var. *moraru*m** **Cuatrec.**, Phytologia 45(1): 25–27. 1980. **TYPE. COLOMBIA: Cauca:** Páramo de Moras, 3600 m, 19 Mar 1973, J.Cuatrecasas & Lehmann 28638 (holotype: US!; isotypes: COL!, US!).

**Common names.** Frailejón (C), frailejón de Moras*.

***Espeletia hartwegiana* var. *vegasan*a** **Cuatrec.**, Phytologia 45(1): 24–25. 1980. **TYPE. COLOMBIA: Valle del Cauca:** Páramo de Las Vegas, in Páramo de Las Hermosas, 3800 m, 22 Mar 1946, J.Cuatrecasas 20285 (holotype: US!; isotypes: COL!, HPUJ!, F!).

**Common names.** Frailejón (C), frailejón de Las Hermosas*.

**3.31 *Espeletia idroboi*** **Cuatrec.**, Phytologia 38(1): 12–15. 1977. **TYPE. COLOMBIA: Cauca:** Valle de Las Papas, 2910 m, Idrobo, Pinto & Bischler 3372 (holotype: COL; isotype: P).

**Common names.** Frailejón (C), frailejón del Cauca*, frailejón de Idrobo (C).

**3.32 *Espeletia incana*** **Cuatrec.**, Revista Acad. Colomb. Ci. Exact. 3(12): 435–436, figs. 20, 23–K, pl. IV. 1940. **TYPE. COLOMBIA: Boyacá:** Páramo de la Rusia, NW slopes, 3500 m, 4 Aug 1940, J.Cuatrecasas 10430 (holotype: COL!; isotypes: COL!, F!, P, US!).

**Common names.** Frailejón (C), frailejón blanco (C), frailejón de los incas*.

***Espeletia incana* f. *prolificen*s** **Cuatrec.**, Revista Acad. Colomb. Ci. Exact. 3(12): 435, figs. 20A–E, 23K. 1940. **TYPE. COLOMBIA: Boyacá:** Páramo de la Rusia, NNW of Duitama, Serranía de Peña Negra, cerca de Las Torres, 3900 m alt, caulirosula 3 m, hojas oscuro-grisaceas, 12 Dec 1972, A.M.Cleef 7124 (Holotype, US!; isotypes: COL!, U)

**Common names.** Frailejón (C).

**3.33 *Espeletia jajoensis*** **Aristeg.**, Fl. Venez. 10(1): 424–425. 1964. **TYPE. VENEZUELA: Trujillo:** El Paramito, between Jajó and Tuñame, 3000 m, Aug 1958, L.Aristeguieta & Medina 3452 (holotype: VEN; isotypes: VEN, US!).

*Espeletiopsis jajoensis* (Aristeg.) Cuatrec., Phytologia 35(1): 56. 1976.

**Common names.** Frailejón (V), frailejón de Jajó*, frailejón peludo blanco (V), frailejón punta de lanza pequeño de hoja ancha (V).

**3.34 *Espeletia jaramilloi*** **S. Díaz**, Mutisia 37: 1–5. 1972. **TYPE. COLOMBIA: Boyacá:** Páramo de Pisba, 3500–3600 m, 9 Oct 1971, R.Jaramillo, G.Lozano & S.Díaz 5047 (holotype: COL; isotype: US!).

**Common names.** Frailejón (C), frailejón de Jaramillo (C).

**3.35 *Espeletia killipii*** **Cuatrec.**, Revista Acad. Colomb. Ci. Exact. 3(12): 425–426, figs. 2, 3, 4–D, E, pl. 1. 1940. **TYPE. COLOMBIA: Cundinamarca:** Páramo de Guasca, eastern slope, 3200–3300 m, 11 Oct 1939, H.García-Barriga 8117 (holotype: COL!; isotype: US!).

**Common names.** Frailejón (C), frailejón de Chisacá*.

***Espeletia killipii* var. *chisacan*a** **Cuatrec.**, Phytologia 32(4): 326. 1975. **TYPE. COLOMBIA: Cundinamarca:** Páramo de Chisacá, 3680–3700 m, 16 Sep 1961, J.Cuatrecasas & Jaramillo 25986 (holotype: US!; isotypes: COL!, F!, MA, P).

**Common names.** Frailejón (C), frailejón de Chisacá*.

**3.36 *Espeletia lopezii*** **Cuatrec.**, Revista Acad. Colomb. Ci. Exact. 3(11): 248–249, figs. 3, 4, 6D, pl. C. 1940. **TYPE. COLOMBIA: Boyacá:** Las Lagunillas, in Nevado del Cocuy, 4100 m, 12 Sep 1938, J.Cuatrecasas & H.García-Barriga 1540 (holotype, COL!; isotypes: F!, P, US!).

**Common names.** Frailejón (C), frailejón perrito (C).

***Espeletia lopezii* subsp. *ursin*a** **Cuatrec.**, *ined*. (Forthcoming publication in Cuatrecasas in press.)

**Common names.** Frailejón del oso*.

***Espeletia lopezii* var. *escobalensi*s** **Cuatrec.**, Phytologia 45(1): 21. 1980. **TYPE. COLOMBIA: Boyacá:** Alto del Escobal, Páramo between Soatá and Cocuy, 3800 m, 8 Sep 1938, J.Cuatrecasas & H.García-Barriga 1236 (holotype: US!; isotypes: COL!, F).

***Espeletia lopezii* var. *majo*r** **Cuatrec.**, Phytologia 31(4): 325–327. 1975. **TYPE. COLOMBIA: Boyacá:** Quebrada del Curial, Nevado del Cocuy, Páramo on the eastern slope, 3350 m, Boyacá, COLOMBIA: 15 Sep 1969, J.Cuatrecasas & Rodríguez 27791 (holotype: COL!; isotype: US!).

***Espeletia lopezii* f. *alticol*a** **Cuatrec.**, Phytologia 31(4): 327–328. 1975. **TYPE. COLOMBIA: Boyacá:** Páramo Cóncavo, Nevado del Cocuy, superPáramo 4335 m, 26 Feb 1973, A.M.Cleef 8547 (holotype: US!; isotypes: COL!, HPUJ!, U).

**3.37 *Espeletia marnixiana*** **S. Díaz & Pedraza**, Revista Acad. Colomb. Ci. Exact. 25(94): 12–15, figs. 3, 4. 2001. **TYPE. COLOMBIA: Cauca:** Mpio. de Argelia, vereda El Naranjal, Río Plateado, Páramo de la Soledad, M.L.Becking 1042 (holotype: COL!).

**Common names.** Frailejón (C), frailejón de Argelia*.

**3.38 *Espeletia marthae*** **Cuatrec.**, Phytologia 38(1): 20–22. 1977. **TYPE. VENEZUELA: Mérida:** Páramo del Guirigay–Llano Corredor, swampy ground, 3300 m, Mérida, VENEZUELA, 25 Oct 1969, J.Cuatrecasas, M.López-Figueiras & Marcano-Berti 28162 (holotype: US!; isotype: MERF!).

**Common names.** Frailejón (V), frailejoncito plateado (V).

**3.39 *Espeletia mirabilis*** **S. Díaz & Rodr.-Cabeza**, Revista Acad. Colomb. Ci. Exact. 34 (133): 441–454, figs. 3F, 4. 2010. **TYPE. COLOMBIA: Boyacá:** Parque Nacional Natural Pisba, Municipio de Socotá, Vereda Corral de Piedra, sector Río Arzobispo, parte alta de Los Estupendos, 3550 m de alt., 05º 58’ 33”N 72º 33’ 47”W, 14 sep 2008, B. V. Rodr.-Cabeza & L. Velasco 2001 (holotype: COL; isotypes: COL!, HUA, UIS, UPTC).

**Common names.** Frailejón (C), frailejón de Los Estupendos*.

**3.40 *Espeletia miradorensis*** **(Cuatrec.) Cuatrec.**, Phytologia 52(3): 158. 1982. *Espeletia grandiflora* var. *miradorensis* Cuatrec., Phytologia 32(4): 324. 1975. **TYPE. COLOMBIA: Cundinamarca:** El Mirador, Páramo de Sumapaz, 3560 m, A.M.Cleef 8421 (holotype: US!; isotypes: COL!, U).

**Common names.** Frailejón (C), frailejón del Mirador*.

**3.41 *Espeletia murilloi*** **Cuatrec.**, Revista Acad. Colomb. Ci. Exact. 3(12): 425–426, Figs. 4-A, B, C, pl. I. 1940b. Ibid. 4(14): 168. 1941. **TYPE. COLOMBIA: Boyacá:** Páramo de Arcabuco, 2800–2950 m, 22 Feb 1940, E.Pérez-Arbeláez & J.Cuatrecasas 8098 (holotype: COL!; isotypes: F!, US!).

*Espeletia murilloi* var. *rusiana* Cuatrec., Revista Acad. Colomb. Ci. Exact. 4(14): 168. 1941. **TYPE. COLOMBIA: Boyacá:** Páramo de La Rusia, 3500 m, 4 Aug 1940, J.Cuatrecasas 10429 (holotype: COL!; isotypes: F!, US!).

*Espeletia murilloi* var. *subcoriacea* Cuatrec., Revista Acad. Colomb. Ci. Exact. 4(14): 168, figs. 11, 12-B, pl. 4. 1941. *Espeletia murilloi* subsp. *subcoriacea* Cuatrec., Revista Acad. Colomb. Ci. Exact. 4(14): 168. 1941. **TYPE. COLOMBIA: Boyacá:** Páramo de la Rusia, 3300–3400 m, 4 Aug 1940, J.Cuatrecasas 10411 (holotype: COL!; isotype: COL).

**Common names.** Frailejón (C), frailejón de Murillo (C).

**3.42 *Espeletia mutabilis*** **S. Díaz & Rodr.-Cabeza**, Revista Acad. Colomb. Ci. Exact. 30 (16): 345–347 figs. 9, 12A–B. 2006. **TYPE. COLOMBIA: Boyacá:** Municipio de Socha, Vereda El Mortiñal (parte alta), sector El Alizal, sitio El Frailejonal, finca de la Alcaldia, 3700 m alt., 28 Jul 2006. B.V.Rodríguez & P.Velasco 1675 (holotype: COL; isotypes: COL!, UIS).

**Common names.** Frailejón (C), frailejón mutante*.

**3.43 *Espeletia nana*** **Cuatrec.**, Phytologia 40(1): 29–31. 1978. **TYPE. VENEZUELA: Trujillo:** La Morita, between Tuñame and Jajó, open páramo, 3000 m, 13 Jul 1971, L.Ruiz-Terán & M.López-Figueiras 2204 (holotype: US!; isotype: MERF!).

**Common names.** Frailejón (V), frailejón enano (V), frailejoncito (V).

**3.44 *Espeletia nemekenei*** **Cuatrec.**, Revista Acad. Colomb. Ci. Exact. 3(12): 430–431 figs. 12, 24-C, pl. II. 1940. **TYPE. COLOMBIA: Boyacá:** Alto de Canutos below Páramo de Guantiva, 3200 m, 3 Aug 1940, J.Cuatrecasas 10348 (holotype: COL!; isotypes: COL!, F!, US!).

**Common names.** Frailejón (C), frailejón de Nemequené (C).

**3.45 *Espeletia occidentalis*** **A. C. Sm.**, Brittonia 1(7): 520–521. 1935. **TYPE. COLOMBIA: Antioquia–Córdoba:** Cordillera Occidental, Páramo de Chaquiro, 3000–3200 m, f.W.Pennell 4266 (holotype: NY!; isotypes: GH, US!).

**Common names.** Frailejón (C), frailejón de occidente*.

***Espeletia occidentalis* subsp. *antioquensi*s** **(Cuatrec.) Cuatrec.**, *ined*. (Forthcoming publication in Cuatrecasas in press.)

*Espeletia occidentalis* var. *antioquensis* Cuatrec.

**Common names.** Frailejón (C), frailejón de Antioquia*.

**3.46 *Espeletia oswaldiana*** **S. Díaz**, Mutisia 32: 1–5, figs. 1, 2. 1970. **TYPE. COLOMBIA: Boyacá:** Vado Hondo, Valle del Río Cusiana, 2880 m, 2 Jul 1968, S.Díaz 74 (holotype COL!; isotype: US!).

**Common names.** Frailejón (C), frailejón de la Sarna*, frailejón de Oswaldo (C).

**3.47 *Espeletia paipana*** **S. Díaz & Pedraza**, Revista Acad. Colomb. Ci. Exact. 25(94): 12–15, figs. 3, 4. 2001. **TYPE. COLOMBIA: Boyacá:** Mpio. de Paipa, Cuchilla El Páramo, 3300 m, D.Stancik 1507 (holotype: COL; isotype: COL!).

**Common names.** Frailejón (C), frailejón de Paipa (C).

**3.48 *Espeletia perijaensis*** **Cuatrec.**, Phytologia 38(1): 17–20. 1977. **TYPE. COLOMBIA: Cesar:** Sierra de Perijá, east of Manaure, 2800 m, 10 Nov 1959, J.Cuatrecasas & R.Romero-Castañeda 25192 (holotype: US!; isotype: COL).

**Common names.** Frailejón (C, V), frailejón de Perijá*.

**3.49 *Espeletia pescana*** **(S. Díaz) S. Díaz**, Revista Acad. Colomb. Ci. Exact. 32 (125): 459. 2008. *Espeletia brachyaxiantha* subsp. *pescana* S. Díaz, Mutisia 61: 8–10, fig. 3. 1985. **TYPE. COLOMBIA: Boyacá:** Páramo La Cortadera, Pesca, 3750 m, 21 Aug 1982, M.Bejarano 257 (holotype: COL; isotype: US!).

**Common names.** Frailejón (C), frailejón de Pesca (C).

**3.50 *Espeletia pisbana*** **S. Díaz & Rodr.-Cabeza**, Revista Acad. Colomb. Ci. Exact. 30 (16): 332–334 figs. 1.4 A–B. 2006. **TYPE. COLOMBIA: Boyacá:** Parque Nacional Natural Pisba. Municipio de Socotá, Vereda Pueblo Viejo, ruta libertadora, sector La Australia, sitio el Alto del Almorzadero, 3406 m alt., 16 jun 2005. B.V.Rodr.-Cabeza & L.Velasco 1389 (holotype: COL; IT; COL!).

**Common names.** Frailejón (C), frailejón de Pisba*.

**3.51 *Espeletia praefrontina*** **Cuatrec.**, Phytologia 47(1): 10–12. 1980. **TYPE. COLOMBIA: Antioquia:** Cordillera Occidental, Páramo de Frontino ca. Llano Grande, J.D. Boeke & J.M. McElroy 273 (holotype: US!; isotype: NY!).

**Common names.** Frailejón (C), frailejón del Sol*.

**3.52 *Espeletia pulcherrima*** **Rodr.-Cabeza & S. Díaz**, Revista Acad. Colomb. Ci. Exact. 30 (16): 339–341 figs. 5, 8A–B. 2006. **TYPE. COLOMBIA: Boyacá:** Municipio de Chita, Vereda Minas, Páramo de Los Venados, carretera hacia Sácama, km 86, desvío a Chita, 3300 m alt., 23 Oct 2005, B.V.Rodríguez-Cabeza & L.Velasco 1520 (holotype: COL; isotype: UIS).

**Common names.** Frailejón (C), frailejón pulcro*.

**3.53 *Espeletia pycnophylla*** **Cuatrec.**, Revista Acad. Colomb. Ci. Exact. 5(17): 24–25, figs. 11-I, 12. 1942. **TYPE. COLOMBIA: Nariño–Putumayo:** Páramo de San Antonio del Bordoncillo (Páramo de Quilinsayaco), 3200 m, 4 Jan 1941, J.Cuatrecasas 11736 (holotype: COL!; isotypes: BC!, F!, US!).

**Common names.** Frailejón (C, E), frailejón del sur*.

***Espeletia pycnophylla* subsp. *angelensi*s** **Cuatrec.**, Phytologia 45(1): 18–20. 1980. **TYPE.** ECUADOR: Carchi: Páramo del Angel, 3400 m, 21 Jun 1939, E.Asplund 7078 (holotype: US!; isotypes: G, K!, US!).

*Espeletia cochensis* Cuatrec., Revista Acad. Colomb. Ci. Exact. 5(17): 25–26, figs. 11J, 13. 1942. **TYPE. COLOMBIA: Putumayo:** Quebrada de Santa Lucía, south side of Laguna de La Cocha, 2850 m, 4 m high, 8 Jan 1941, J.Cuatrecasas 11820 (holotype: COL!; isotypes: F!, US!).

**Common names.** Frailejón (C, E), frailejón del sur*.

***Espeletia pycnophylla* subsp. *llanganatensi*s** **Cuatrec.**, Phytologia 45(1): 20–21. 1980. **TYPE.** ECUADOR: Tungurahua: Cordillera de los Llanganates, near Las Torres, 3700–3800 m, 23 Nov 1939, E.Asplund 9944 (holotype: S).

**Common names.** Frailejón (E), singurima (E).

***Espeletia pycnophylla* var. *galeran*a** **Cuatrec.**, Phytologia 45(1): 17. 1980. **TYPE. COLOMBIA: Nariño:** Volcán Galeras, Páramo 3900 m, 7 Feb 1965, J.Cuatrecasas & L.E.Mora 26931 (holotype: US!; isotype: COL).

***Espeletia pycnophylla* var. *lacinulat*a** **Cuatrec.**, Phytologia 45(1), 17–18. 1980. **TYPE. COLOMBIA: Nariño:** Páramo de Quilinsayaco, 3200–3250 m, 21 Mar 1973, J.Cuatrecasas, E.Hernández & A.Estrada 28653 (holotype: US!; isotypes: COL!, PASTO).

**3.54 *Espeletia raquirensis*** **S. Díaz & Rodr.-Cabeza**, Revista Acad. Colomb. Ci. Exact. 34 (133): 441–454, fig. 2, 3C–D. 2010. **TYPE. COLOMBIA: Boyacá:** Municipio de ráquira, Vereda Firita, Peña Arriba, Páramo de Rabanal, en límites con el Municipio de Guachetá, vereda San Antonio (Cundinamarca), 3200 m alt., 05º 24’N 73º 36’W, 12 Aug 2008, B.V.Rodríguez-Cabeza, R.Galindo-T, & I.Cortez 1973 (holotype: COL; isotypes: COL!, HUA, UIS, UPTC).

**Common names.** Frailejón (C), frailejón de Ráquira*.

**3.55 *Espeletia robertii*** **Cuatrec.**, Phytologia 38(1): 7–10. 1977. **TYPE. COLOMBIA: Norte de Santander–Cesar:** linea divisoria, Cerro de Las Juridicciones, H.García-Barriga & R.Jaramillo M. 20648 (holotype: US!; isotype: COL!).

**Common names.** Frailejón (C), frailejón de Jurisdicciones (C), frailejón de Oroque*, frailejoncito (C).

**3.56 *Espeletia rositae*** **Cuatrec.**, Revista Acad. Colomb. Ci. Exact. 4(14): 164–165, figs. 5, 12I, pl. III. 1941a. Ibid. 4(15–16): 341. 1941. **TYPE. COLOMBIA: Boyacá:** Páramo de Santa Rosita 3300–3400 m, 3 Aug 1940, J.Cuatrecasas 10371 (holotype: COL!; isotypes: COL!, F!, US!).

**Common names.** Frailejón (C), frailejón de Santa Rosita (C).

***Espeletia rositae* subsp. *macrocephal*a** **(Cuatrec.) Cuatrec.**, *ined*. (Forthcoming publication in Cuatrecasas in press.)

*Espeletia rositae* var. *macrocephala* Cuatrec.

**Common names.** Frailejón (C), frailejón de Santa Rosita (C).

**3.57 *Espeletia schultesiana*** **Cuatrec.**, Revista Acad. Colomb. Ci. Exact. 5(17): 26–27, figs. 14, 11F, G, H, pl. II. 1942. **TYPE. COLOMBIA: Putumayo:** Páramo del Tambillo, 2700–2800 m, 13–14 Dec 1941, R.E.Schultes & C.E.Smith 3096 (holotype: COL!; isotypes: BR, C, COL!, F!, F, FI, G, GH, HPUJ!, MO!, NCU, NY!, P, U!, US!, W).

**Common names.** Frailejón (C), frailejón de Schultes (C), frailejón “ush” (C, Kamsá).

***Espeletia schultesiana* f. *alternifoli*a** **(Cuatrec.) Cuatrec.**, *ined*. (Forthcoming publication in Cuatrecasas in press.)

*Espeletia alternifolia* Cuatrec.

**Common names.** Frailejón (C).

**3.58 *Espeletia schultzii*** **Wedd.**, Chlor. And. 1: 63–64. 1855. **TYPE. VENEZUELA: Mérida:** Páramos prov. Mérida, 3300–3600 m, Jun 1842, J.J.Linden 370, lectotype P; isotypes: BM, BR, F!, FI, G, LE, P, US!, W).

*Espeletia corymbosa* Sch. Bip. ex Wedd., nom. illeg. Chlor. And. 1: 63. 1856 [1855 publ. 30 Jun 1856].

**Common names.** Frailejón (V), frailejón amarillo (V), frailejón común (V), frailejón lanudo (V), frailejón manso (V), frailejón octubre (V).

***Espeletia schultzii* var. *bractilobat*a** **Cuatrec.**, Phytologia 45(1): 27. 1980. **TYPE. VENEZUELA: Trujillo:** Paramillo above Jajó via Tuñame, 3100 m, 29 Oct 1969, J.Cuatrecasas, L.Ruiz-Terán & M.López-Figueiras 28189 (holotype: US!; isotype: MERF!).

***Espeletia schultzii* var. *mucuruban*a** **Cuatrec.**, Phytologia 45(1): 28. 1980. **TYPE. VENEZUELA: Mérida:** Páramo de Mucurubá, 3250 m, 20 Oct 1969, J.Cuatrecasas, L.Ruiz-Terán & M.López-Figueiras 28148 (holotype: US!; isotypes: MERF!).

***Espeletia schultzii* var. *subparamun*a** **Cuatrec.**, Phytologia 45(1): 29. 1980. **TYPE. VENEZUELA: Trujillo:** Páramo de La Cristalina, 2500–2600 m, 17 Feb 1973, J.Cuatrecasas, L.Ruiz-Terán & M.López-Figueiras 28557 (holotype: US!; isotypes: BC!, F!, G!, MERF!, NY!, US!).

**Note.** Specimens of this taxon have been annotated with the name *Espeletia cristalina* Cuatrec. However, this combination was never published.

**3.59 *Espeletia semiglobulata*** **Cuatrec.**, Ciencia (Mexico) 6(7–9): 264, fig. 5. 1945. **TYPE. VENEZUELA: Mérida:** Páramo de Piedras Blancas, 3800 m, V.M.Badillo 821 (holotype: VEN; isotype: VEN).

*Espeletia oppositifolia* Sch. Bip., mscr. pro parte ex Wedd., Chlor. And. 1: 62. 1855. *Nomen nudum*.

*Espeletia rufescens* Cuatrec., Bol. Soc. Venez. Ci. Nat. 17 (85): 88–91, figs. 5, 6. 1956. **TYPE. VENEZUELA: Mérida:** Sierra Nevada de Mérida, 12000 ft, J.J.Linden 398, (lecto-holotype: P, only inflorescence; J.J.Linden 399 paratype: P, pro parte, only inflorescence).

**Common names.** Frailejón (V), frailejón semigloboso*.

**Note.** *Espeletia rufescens* is an herbarium mixture and only the synflorescences of the type collection of *Espeletia rufescens* correspond to *Espeletia semiglobulata* (see *Espeletia rufescens* below).

**3.60 *Espeletia soroca*** **S. Díaz & Rodr.-Cabeza**, Revista Acad. Colomb. Ci. Exact. 30 (16): 334–336 figs. 2, 4C–D. 2006. **TYPE. COLOMBIA: Boyacá:** Municipio de Chita, Vereda Minas, Páramo de Los Venados, carretera hacia Sácama, km 86, desvío a Chita. 3200 m **alt.,** 23 oct 2005. B. V. Rodr.-Cabeza & L. Velasco 150 (holotype: COL; isotype: COL!).

**Common names.** Frailejón (C), frailejón soroco*.

**3.61 *Espeletia standleyana*** **A. C. Sm.**, Brittonia 1(7): 514–515, Pl. I, 17–21. 1935. **TYPE. COLOMBIA: Santander:** Páramo de Santurbán, 3800 m, E.P.Killip & A.C.Smith 19558 (holotype: NY!; isotypes: COL!, GH, P, PHIL, US!).

**Common names.** Frailejón (C), frailejón de Bucaramanga*, frailejón de Standley (C).

***Espeletia standleyana* subsp. *ampl*a** **(Cuatrec.) Cuatrec.**, *ined*. (Forthcoming publication in Cuatrecasas in press.)

*Espeletia standleyana* var. *ampla* Cuatrec.

***Espeletia standleyana* subsp. *laxio*r** **(Cuatrec.) Cuatrec.**, *ined*. (Forthcoming publication in Cuatrecasas in press.)

*Espeletia standleyana* var. *laxior* Cuatrec.

**3.62 *Espeletia steyermarkii*** **Cuatrec.**, Ciencia (México) 6(7–9): 265–266, fig. 6. 1945. **TYPE. VENEZUELA: Táchira:** Quebrada del Palmar, 2500 m, Páramo de Tamá, J.Steyermark 57217 (holotype: VEN; isotypes: F!, NY!, US!).

*Espeletia brassicoidea* var. *contracta* sensu Aristeg., Fl. Venez. 10(1): 449, 451. 1964.

**Common names.** Frailejón (C, V), frailejón ramoso*.

**3.63 *Espeletia summapacis*** **Cuatrec.**, Phytologia 31(4): 331–333. 1975. **TYPE. COLOMBIA: Cundinamarca:** Macizo de Sumapaz, near Pico de San Mateo, 3950–4000 m, 7 Feb 1975, L.Uribe-Uribe & R.Jaramillo 6895 (holotype: US!; isotypes: COL!, HPUJ!, G!).

**Common names.** Frailejón (C), frailejón de Sumapáz*.

**3.64 *Espeletia tapirophila*** **Cuatrec.**, Phytologia 32(4): 320–322. 1975. **TYPE. COLOMBIA: Cundinamarca:** Puerta de las Dantas, Páramo de Sumapaz, 3400 m, A.M.Cleef 8301 (holotype: US!; isotypes: COL!, U).

**Common names.** Frailejón (C), frailejón de las dantas (C).

**3.65 *Espeletia tenorae*** **Aristeg.**, Bol. Soc. Venez. Ci. Nat. 20(93): 275–279. 1959. **TYPE. VENEZUELA: Trujillo:** Páramo del Guirigay, ca. Laguna La Parida, 3500 m, Aug 1958, L.Aristeguieta & Medina 3572 (holotype: VEN; isotypes: NY!, US!).

**Common names.** Frailejón (C), frailejoncito (C), frailejoncito de Guirigay*.

**3.66 *Espeletia tibamoensis*** **S. Díaz & Rodr.-Cabeza**, Revista Acad. Colomb. Ci. Exact. 34 (133): 441–454, figs. 3E, 5. 2010. **TYPE. COLOMBIA: Boyacá:** Límites entre los municipios de Siachoque y Toca, veredas Cormechoque arriba y Tubenecos, Páramo La Cortadera, sector Alto Tibamoa, 3600 m alt, 16 May 2008, B.V.Rodr.-Cabeza & A.Burgos 1959. (holotype: COL; Isotypes: COL, HUA, UIS, UPTC).

**Common names.** Frailejón (C), frailejón de Tibamoa*.

**3.67 *Espeletia tillettii*** **Cuatrec.**, Phytologia 47(1): 8–10. 1980. **TYPE. VENEZUELA: Zulia:** Sierra de Perijá, 3100 m, S.S.Tillett 747–1126 (holotype: US!; isotypes: MYF!, VEN).

**Common names.** Frailejón (C, V), frailejón de Zulia*.

**3.68 *Espeletia tunjana*** **Cuatrec.**, Revista Acad. Colomb. Ci. Exact. 3(12): 433–434, figs. 16, 23H, G, pl. III. 1940. **TYPE. COLOMBIA: Boyacá:** Páramo de Santa Rosa de Viterbo, El Portachuelo, 3000 m, 3 Aug 1940, J.Cuatrecasas 10338 (lectotype: COL; isotypes: F!, US!; photo of holotype (Cuatrecasas C-1727): US!).

**Common names.** Frailejón (C), frailejón de Tunja (C).

**3.69 *Espeletia ulotricha*** **Cuatrec.**, Phytologia 23(4): 364–365. 1972. **TYPE. VENEZUELA: Lara–Trujillo:** Páramo del Jabón, 3000 m, in open grass Páramo, 2 Nov 1969, J.Cuatrecasas, L.Ruiz-Terán & M.López-Figueiras 28220 (holotype: P; isotypes: MERF!, US!).

**Common names.** Frailejón (V), frailejón salchicha*, frailejoncito (V).

**3.70 *Espeletia uribei*** **Cuatrec.**, Mutisia 16: 1–2, fig. 1. 1953. Ibid. 19: 9. 1954. **TYPE. COLOMBIA: Cundinamarca:** Páramo de La Siberia mun. La Calera 3500 m, 26 Oct 1952, L.Uribe-Uribe 2475 (holotype: F!; isotypes: P, US!).

**Common names.** Frailejón (C), frailejón de Chingaza*.

**3.71 *Espeletia weddellii*** **Sch. Bip. ex Wedd.**, Chlor. And. 1: 66, Pl. 15-B. 1856. **TYPE. VENEZUELA: Trujillo:** Páramo de Niquitao, La Teta, 4000 m, Jul 1843, J.J.Linden 1443 (lectotype: P; lectoisotypes: BR, F!, FI, G, K!, LE, US!, W).

**Common names.** Chijí (V), chijí chiquito (V), frailejón (V), frailejón batata (V), frailejón chijí (V), frailejón casco de burro (V), frailejón pata de burro (V).

**Hybrids**

***Espeletia ×garcibarrigae*** **Cuatrec. (= *Espeletia argentea* f. *phaneractis* (S. F. Blake) Cuatrec.** × ***Espeletia grandiflora* H. & B.)**, Revista Acad. Colomb. Ci. Exact. 3(12): 426–427. 1940. **TYPE. COLOMBIA: Cundinamarca:** Páramo de Guasca, 3000–3500 m alt., 2 Oct 1939 H.García-Barriga 08108 (holotype: COL).

*Espeletia ×pachoana* Cuatrec., Revista Acad. Colomb. Ci. Exact. 4(14): 165. figs. 6, 12E, tab. 4. 1941. **TYPE. COLOMBIA: Cundinamarca:** Cordillera Oriental, Páramo de Zipaquirá, entre Zipaquirá y Pacho, 3100–3200 m alt., 16 Jun de 1940, J.Cuatrecasas 9563 (holotype: COL; isotype: US!).

*Espeletia ×pachoana* f. *brevifolia* Cuatrec., Revista Acad. Colomb. Ci. Exact. 4(14): 166. 1941. **TYPE. COLOMBIA: Cundinamarca:** Cordillera Oriental, Páramo de Zipaquirá, entre Zipaquirá y Pacho, 3100–3200 m alt., 16 Jun de 1940, J.Cuatrecasas 9561 (holotype: COL; isotype: US!).

***Espeletia ×verdeana*** **Cuatrec. (= *Espeletia argentea* H. & B.** × ***Espeletia grandiflora* H. & B.)**, Revista Acad. Colomb. Ci. Exact. 4(14): 166, figs. 8, 12F. 1941. **TYPE. COLOMBIA: Cundinamarca:** Cordillera Oriental, Páramo de Cruz Verde, 3400–3500 m alt., 15 Sep 1940, J.Cuatrecasas 10477 (holotype: COL; isotype: US!).

***Espeletia ×guascensis*** **Cuatrec. (= *Espeletia argentea* f. *phaneractis*** × ***Espeletia killipii* Cuatrec.)**, Revista Acad. Colomb. Ci. Exact. 4(14): 166, figs. 7, 12K. 1941. **TYPE. COLOMBIA: Cundinamarca:** Páramo de Guasca, Cordillera Oriental, 3200–3300 m, 2 Jun 1940, J.Cuatrecasas 9493 (holotype: COL!; isotypes: COL!, US!).

***Espeletia batata* Cuatrec. *× E. schultzii* Wedd.** **Representative specimens. VENEZUELA: Mérida:** páramo de Los Granates, Sierra nevada de Santo Domingo, Alto del Morato, 3670 m, rósula, hojas blancas o blanco verdosas, escapos ramosos con 3–5 capítulos, 12 Oct 1969, J.Cuatrecasas 28083 (US!); ibidem: Páramo de Piedras Blancas, después de las Cruces, carretera vía Piñango, 3600 m, subfrútice más pequeño que *Espeletia schultzii* y más grande que *Espeletia batata*, con hábitos morfológicamente intermedios entre esas dos especies, en general presenta más de 3 inflorescencias por eje, de colores llamativos (amarillo), 22 Sep 1984, J.M.Moreno-Alvarez 170 (US!); ibidem: Rangel, Páramo de Mucuchíes, alredores de la Piedra Grietada, junto a al carretera hacia la población de Piñango, cordillera de los Andes, 4300 m, acaulirroruleto de 50–55 cm de altura total, roseta foliar de 40 cm de alto x 26 cm de diámetro, hojas oblanceoladas, crespo-lanosas en ambas caras, los pelos blanquecinos, hojas tiernas con pelos blancos, muy largos, seríceos, que se entrelazan con los de las hojas adyacentes, formando a modo de una telaraña cuando se mira la roseta desde arriba, ejes inflorescenciales alrededor de 7, axilares, erectos, cimoso-ramificados distalmente, cilíndricos con indumento lanoso, denso marrón, presenta también en el involucro, capítulos heterógamo-radiados, péndulos, 4 cm de diámetro total y 13 mm en el disco, lígulas numerosas, suberectas, intensamente amarillas, flósculos amarillos, 18 Nov 1970, L.Ruiz-Terán 1081 (US!, F!).

***Espeletia conglomerata* Cuatrec.** × ***Espeletia brassicoidea*** **Cuatrec.**

*Espeletia conglomerata* var. *macroclada* Cuatrec., Revista Acad. Colomb. Ci. Exact. 5(17): 23. 1942. **TYPE. COLOMBIA: Santander:** Páramo del Almorzadero northern slopes, 3600–3800 m, 28 Nov 1941, J.Cuatrecasas 13494A (holotype: COL; isotypes: F!, US!).

*Espeletia conglomerata* var. *pedunculata* Cuatrec., Revista Acad. Colomb. Ci. Exact. 5(17):23. 1942. **TYPE. COLOMBIA: Santander:** Páramo del Almorzadero, eastern slopes, 3600–3800 m, 28 Nov 1941, J.Cuatrecasas 13494 (holotype: COL; isotypes: F!, US!).

**Note.** There are a few scattered individuals of this hybrid in the topolocality, between the abundant populations of *Espeletia brassicoidea* and *Espeletia conglomerata*. *Espeletia brassicoidea* is found generally in lower elevations than *Espeletia conglomerata*, which is more abundant in the Páramo del Almorzadero, especially on the north side. The hybrid presents different levels of introgression, being *Espeletia conglomerata*-dominant in its morphology. In some specimens (e.g. those corresponding originally to *Espeletia conglomerata* var. *macroclada*) the synflorescences are more similar to those of *Espeletia brassicoidea*, while the narrowly oblong leaves are typical of *Espeletia conglomerata*. The short tube of the ray corollas also matches with those in *Espeletia conglomerata*. In other specimens (e.g. those identified as *Espeletia conglomerata* var. *pedunculata*) the general appearance of the synflorescence is similar in shape and size to *Espeletia conglomerata*, with regular dense cymes, but with a pair of two long straight peduncles at the base, probably as an introgression with *Espeletia brassicoidea* in which the first two pairs of peduncles are well developed.

***Espeletia congestiflora* Cuatrec.** × ***Espeletia murilloi*** **Cuatrec.** **Representative specimens. COLOMBIA: Boyacá:** Páramo de La Rusia, NW–N de Duitama: aislada, cerca del empalme de la carretara, 3540 m, acaulirósula hojas grisáceas, lígilas amarillas, 13 Dec 1972, A.M.Cleef 7184a (US!, MO!).

***Espeletia conglomerata* Cuatrec.** × ***Espeletia dugandii*** **Cuatrec.** **Representative specimens. COLOMBIA: Norte de Santander:** between Pamplona and Malaga, on highway, arborescent, 6 inches to 4 feet high, stem diameter 6 inches or more, photo: SI 3243, used to make cushions and mattresses, 24 Mar 1935, W.A.Archer 3243 (US).

***Espeletia grandiflora* H. & B.** × ***Espeletia killipii*** **var. *chisacana* Cuatrec.** **Representative specimens. COLOMBIA, CUNDINAMARCA:** Páramo de Sumapaz, Chisacá near the road-marker 30, 3700 m, caulirosula 1 m, associated with *Puya*, *Calamagrostis* and *Arcytophyllum*, 5 Aug 1974, Grabant & J.Idrobo 86 (U, US!); ibidem: Páramo de Chisacá, 17 Mar 1970, R.E.Schultes & I.Cabrera 26009 (GH).

***Espeletia schultzii* Wedd.** × ***Coespeletia moritziana*** **(Sch. Bip. ex Wedd.) Cuatrec.** **Representative specimens. VENEZUELA: Mérida:** Rangel, vía El Aguila–Piñango, entre la Quebrada de Mifafí y la mina abandonada de caolín, 4170 m, caulirrósula de ca 1 m de altura, tallo de 50 cm Hojas con pelos blancuzcos, lanosos, sinflorescencias axilares de ca 1 m de altura, con 3–5 capítulos por eje, dispuestos en forma racemosa, pedúnculo erecto en su base y recurvado en su ápice, capítulos más o menos en plano vertical o algo nutantes, lígulas amarillas en la base, rojizas en el ápice, estilos morado oscuro, flores del disco moradas con polen amarillo, 5 Sep 1983, P.E.Berry 4188 (US!, MO!); ibidem: Páramo de las Cruces, a 6.3 km de El Aguila, del otro lado de la fila, 4250 m, monocaule arrosetada sin tronco evidente, sinflorescencias axilares con racimos de capítulos semi-péndulos, lígulas amarillas teñidas de morado, estilos morados, 9 Sep 1983, P.E.Berry, 4200 (US!, MO!), ibidem: a 6.3 km de El Aguila, del otro lado de la fila, 4250 m, monocaule arrosetada sin tronco evidente, sinflorescencias axilares con racimos de capítulos semi-péndulos, lígulas amarillas teñidas de morado, estilos morados, disco pardo–morado, polen amarillo, 9 Sep 1983, P.E.Berry 4201 (US!, MO!).

***Espeletia schultzii* Wedd.** × ***Coespeletia spicata* (Sch. Bip. ex Wedd.) Cuatrec.** **Representative specimens. VENEZUELA: Mérida:** Rangel, base del Pico de Piedras Blancas, 4000 m, caulirrósula de 1 m de alto, parte aérea del tallo poco desarrollada, vaina foliar marrón clara, lígulas cortas, amarillas, 15 Sep 1984, P.E.Berry 4379 (US!).

***Espeletia schultzii* Wedd. *× Coespeletia timotensis* (Cuatrec.) Cuatrec.** **Representative specimens. VENEZUELA: Mérida:** Justo Briceño, Páramo y chirivital en la vertiente NW del Alto del Totumo, hoya del Río Chirurí, a 19.5 km de El Aguila por la carretera a Piñango, 3900–4000 m, caulirrósula de 1/2 m de alto, sinflorescencias axilares, racemosas, capítulos levemente inclinados hacia abajo, lígulas amarillas, estigmas rojo–morados, flores del disco amarillas, 8 Jan 1983, P.E.Berry 3999 (US!, MO!); ibidem: sinflorescencias axilares, ramosas, capítulos divergentes, lígulas amarillas, flores del disco amarillas, estigmas morados, 12 Oct 1983, P.E.Berry 4233 (US!, MO!); ibidem: Miranda, páramo en las cabeceras de la Quebrada El Turmero (afluentes del Río Motatán), a 6.3 km de El Aguila por la carretera a Piñango, 4280 m, caulirrósula de 1 m de alto, sinflorescencias axilares, dicasiales, capítulos orientados hacia el lado, lígulas amarillas por el haz, rojo–anaranjado por el envés, estigmas morados, polen amarillo, 12 Nov 1984, P.E.Berry 4398 (US!, MO!).

***Espeletia ×algodonosa*** **Aristeg.** (= ***Espeletia schultzii* Wedd.** × ***Espeletia nana*** **Cuatrec.)**, Bol. Soc. Venez. Ci. Nat. 20(93): 282–283, figs., 1959a. **TYPE. VENEZUELA: Trujillo:** El Paramito, toward Tuñame, Jajó–La Morita Rd, 3000 m, Aug 1958, L.Aristeguieta & Medina 3543 (holotype: VEN; isotypes: NY!, US!); additional collections: **VENEZUELA: Trujillo:** Páramo de Tuñame, El Paramito, un sector del Páramo de Tuñame, vía Jajó–La Morita, 3000 m, planta arrosetada, cabezuelas amarillas, 1 Aug 1958, L.Aristeguieta 3449 (US!); ibidem: Páramo de Cabimbú, alrededores de la Teta de Niquitao, un sector del páramo de Cabimbú, 4000 m, planta acaule de 25–30 cm de altura, ejes inflorescenciales axilares, ramificados, capítulos con lígulas amarillas, 2 Feb 1976, M.López-Figueiras 11883 (US!); ibidem: Mérida: Rangel, Páramo de Guirigay, Llano Corredor, 3000 m, hierba acaule hojas de 27 cm x 2–3 cm de largo, vaina de 2.5 x 2.6 cm, inflorescencias axilares ca de 78 cm de largo, capítulos con lígulas amarillas, 16 Oct 1972, M.López-Figueiras 8868 (US!).

**Note.** Specimens of this proposed hybrid have been collected at El Paramito, between Jajó and Tuñame, and in the páramo of Guirigay–Teta de Niquitao. *Espeletia nana* and *Espeletia schultzii* have large sympatric populations in both localities. The morphological characteristics (e.g. size and shape of leaves and synflorescences) of the specimens of *Espeletia* ×algodonosa are intermediate between the parental species, with different levels of introgression.

***Espeletia schultzii* Wedd. *× Ruilopezia lindenii* (Sch. Bip. ex Wedd.) Cuatrec.** **Representative specimens. VENEZUELA: Mérida:** Campo Elías, páramo de San José, vía a Mucutuy, a 6.5 km de San José, 2970 m, hierba arrosetada, con varias rosetas juntas, sinflorescencia terminal, racemosa, lígulas y flores del disco amarillas, 22 Aug 1984, P.E.Berry 4358 (US!).

***Espeletia tenorae* Aristeg.** × ***Espeletia schultzii*** **Wedd.** **Representative specimens. VENEZUELA: Mérida:** Páramo de Guirigay, Llano Corredor, camino del Arenal, 3200 m, acaulirrosuleto, inflorescencias axilares, capítulos con lígulas amarillas, 10 Jan 1978, M.López-Figueiras, 14517 (US!).

***Espeletia weddellii* Sch. Bip. ex Wedd.** × ***Espeletia marthae*** **Cuatrec.** **Representative specimens. VENEZUELA: Mérida:** Llano corridor between Las Piedras and Guirigay, 3300 m, 25 Oct 1969, J.Cuatrecasas, M.López-Figueriras & L.Marcano-Berti 28163 (holotype: US!; isotypes: US!, MERF).

***Espeletia weddellii* Sch. Bip. ex Wedd. *× Espeletia schultzii* Wedd.** **Representative specimens. VENEZUELA: Mérida:** Páramo de Los Granates, near Laguna Brava, 3300 m, 20 May 1971, M.López-Figueriras 8725 (holotype: US!; isotypes: US!, MERF).

**Herbarium mixture**

***Espeletia rufescens*** **Cuatrec. (= *Coespeletia moritziana* (Sch. Bip. ex Wedd.) Cuatrec. (leaves) + *Espeletia semiglobulata* Cuatrec. (inflorescences))**, Bol. Soc. Venez. Ci. Nat. 17(85): 88, figs. 5–6. 1956. **TYPE. VENEZUELA: Mérida:** Sierra Nevada de Mérida, 12000 ft, J.J.Linden 398 (lectoholotype: P; J.J.Linden 399 paratype: P).

**Note.** As suggested by Weddell (1855[1857]: 64-65) and later by Cuatrecasas (in press), the two mounted specimens that exist (Linden 398 and 399) show clearly the leaves from *Coespeletia moritziana*, except for one that is probably from *Espeletia semiglobulata*, and the synflorescences from *Espeletia semiglobulata*.

**4 *Espeletiopsis*** **Cuatrec.**, Phytologia 35(1): 54–56. 1976. **TYPE.** *Espeletiopsis jimenez-quesadae* (Cuatrec.) Cuatrec.

**4.01 *Espeletiopsis angustifolia*** **(Cuatrec.) Cuatrec.**, Phytologia 35(1): 55. 1976. *Espeletia angustifolia* Cuatrec., Bol. Soc. Venez. Ci. Nat. 17 (85): 80. 1956. **TYPE. VENEZUELA: Mérida:** Páramo de Mijara, 3300 m alt.; 18 Mar 1922, A.Jahn 973 (holotype: US!; isotype: G!).

**Common names.** Frailejón (V), frailejón blanco (V), frailejón plateado (V), frailejón plateado de Mérida*.

**4.02 *Espeletiopsis betancurii*** **Rodr.-Cabeza, S. Díaz & Gal.-Tar.**, Revista Acad. Colomb. Ci. Exact. 30 (16): 349–352 figs. 11, 12C–D. 2006. **TYPE. COLOMBIA: Boyacá:** Municipio de Chita, Vereda Minas, Páramo de Los Venados, carretera hacia Sácama, km 86 desvío a Chita, 3200 m alt., 23 Oct 2005. B.V.Rodr.-Cabeza & L.Velasco 1515 (holotype: COL; isotype: UIS).

**Common names.** Frailejón (C), frailejón de los venados*.

**4.03 *Espeletiopsis caldasii*** **(Cuatrec.) Cuatrec.**, Phytologia 35(1): 55. 1976. *Espeletia caldasii* Cuatrec., Revista Acad. Colomb. Ci. Exact. 3(11): 431. 1940. **TYPE. COLOMBIA: Norte de Santander:** Páramo de Santurbán, extremo occidental, 3400 m alt., 27 Aug 1940, J.Cuatrecasas & H.Garcia-Barriga 10317 (holotype: US).

**Common names.** Frailejón (C), frailejón enano (C), frailejón enano de Caldas*, frailejoncito (C).

**4.04 *Espeletiopsis colombiana*** **(Cuatrec.) Cuatrec.**, Phytologia 35(1): 55. 1976. *Espeletia colombiana* Cuatrec., Revista Acad. Colomb. Ci. Exact. 3(11): 249. 1940. **TYPE. COLOMBIA: Boyacá:** Cordillera Oriental, Nevado del Cocuy; Las Lagunillas, Pozo Azul; páramo 4.110 m alt., 11 Sep 1938, J.Cuatrecasas & H.García-Barriga 1432 (holotype: US!; isotype: F!).

**Common names.** Frailejón (C), frailejón colombiano*.

**4.05 *Espeletiopsis corymbosa*** **(Humb. & Bonpl.) Cuatrec.**, Phytologia 35(1): 55. 1976. *Espeletia corymbosa* Humb. & Bonpl., Pl. Aequinoc. 2: 16. 1809. **TYPE. COLOMBIA: Cundinamarca:** Nous avons trouvé l’Espeletia corymbosa dans la Cordillére des Andes, prés la ville d’Alamaguer, á 1163 toises (2268 métres) d’élévation au-dessus du niveau de l’Océan, c’est le seul endroit oú nous ayons recontré cette plante, elle s’éléve á cinq á six pieds (2 métres) de hauteur, et fournit une aussi grande quantité de résine que l’Espeletia grandiflora, ces deux plantes pourroient donc etre cultivées en Amérique et en Europe, et la résine qu’elles produisent seroit employée utilement dans les arts (from a Cuatrecasas draft: “Type: Humboldt & Bonpland in ‘Herb. H.B.K.’ holotype, ficha No. ? ‘Cordillera los Andes pre la Ville d’Almaguer a 1163 toises (2268 m) elevation.’”), (holotype: P).

*Espeletia corymbosa* var. *foliosa* Duse, Nuovo Giorn. Bot. Ital., Nuova ser. 12: 294. 1905. **TYPE. COLOMBIA: Cundinamarca:** Neuva Grenada: Bogota, locis frigidis, Goudot, Mar 1844.

*Espeletia corymbosa* subsp. *zipaquirana* Cuatrec., Revista Acad. Colomb. Ci. Exact. 4: 168, figs. 10, 12a, tab. 3. 1941. **TYPE. COLOMBIA: Cundinamarca:** Páramo de Zipaquirá, 3100–3200 m, 16 Jan 1940, J.Cuatrecasas 9564 (holotype: F; isotypes: COL!, US!, MA!).

*Espeletia rigida* Humb. & Bonpl., Pl. Aequinoc. 2(9): t. 72. 1808 [1809 publ. Nov 1808].*Nomen nudum*.

*Espeletia platylepis* Sch. Bip. ex Wedd., Chlor. And. 1: 64. 1856 [1855 publ. 30 Jun 1856]. **TYPE. NOUVELLE GRENADE: Cordillères de Bogota:** h. 2250 mètres, Goudot & J.J.Linden 1291; environs d’Almaguer!, h. 2050 mètres, Humb. & Bonpl.

**Common names.** Frailejón (C), frailejón liso*, oreja de mula (C).

**4.06 *Espeletiopsis funckii*** **(Sch. Bip. ex Wedd.) Cuatrec.**, Phytologia 35(1): 55. 1976. *Espeletia funckii* Sch. Bip. ex Wedd., Chlor. And. 1: 64. 1856 [1855 publ. 30 Jun 1856]. **TYPE. COLOMBIA: Norte de Santander:** Hab. Nouvelle grenade: Andes de Pamplona, à une élévation de 3400 mètres, Jan 1847, V.A.Funck & L.J.Schlim 1290 (holotype: P; isotypes: F, G!).

*Espeletia smithiana* Cuatrec., Revista Acad. Colomb. Ci. Exact. 4(14): 339, figs. 3, 4. 1941. **TYPE. COLOMBIA: Santander:** Cordillera Oriental: Páramo de Santurbán entre Cuesta Boba y el extremo oeste, 3400 m alt., 27 Jul 1940, J.Cuatrecasas & H.García-Barriga 10315 (holotype: COL!; isotypes: COL!, F!, HPUJ!, P, US!).

**Common names.** Frailejón (C), frailejón de Funk (C), frailejón de oro*.

**Note.** *Espeletia smithiana* was not included in the last publications of Cuatrecasas (Cuatrecasas 1976; Cuatrecasas 1996; Cuatrecasas in press), probably because he was uncertain regarding the position of this species. Certainly it was an *Espeletiopsis*, although he never published the new combination. The comparison between numerous specimens of *Espeletia funckii* and *Espeletia smithiana* shows no difference between the two. High morphological variation between specimens suggests also that this species might have frequent hybridization.

**4.07 *Espeletiopsis garciae*** **(Cuatrec.) Cuatrec.**, Phytologia 35(1): 55. 1976. *Espeletia garciae* Sch. Bip. ex Wedd., Phytologia 23(4): 358–360. 1972. Et in Ciencias 27(6): 176. 1972. **TYPE. COLOMBIA. Boyacá:** Leiva, vereda de Capilla, encima del km 21, en el monte, 2640 m alt., tallo liso de 4 m alt × 6 cm diám que culmina en tres cortas ramificaciones, flores amarillas, 2 Dec 1970, L.Uribe-Uribe 6491 (holotype: US!; isotypes: MO!); ibidem: vereda de Capilla, monte sobre km 21, 2640 m, mata 6 m, tallo muy duro bifucado a 1 m sobre el suelo, cada rama terminada en cortas ramificaciones apicales, inflorescencias erguidas con flores amarillas, crece en matorral alto, 2 Dec 1970, L.Uribe-Uribe 6492 (paratype: US!); ibidem: Arcabuco, al NE de la poblacion, cerca límite con Santander, 2650 m, 4 m, erecta, tallo 5 cm diam, nervios foliares color oro, pelos amarillos, flores amarillas, cuando se corta se ramifica, 12 Oct 1966, H.García Barriga 18764 (paratype: US!).

**Common names.** Frailejón (C), frailejón de Arcabuco*, frailejón de García-Barriga (C).

**4.08 *Espeletiopsis guacharaca*** **(S. Díaz) Cuatrec.**, Phytologia 35(1): 55. 1976. *Espeletia guacharaca* S. Díaz, Caldasia 11(53): 19. 1975. *Espeletiopsis jimenez-quesadae* var. *guacharaca* (S. Díaz) Cuatrec., Anales Jard. Bot. Madrid 54(1): 374. 1996. **TYPE. COLOMBIA: Boyacá:** Páramo de la Rusia, cerca a la cima, carretera Duitama–Charalá, 3.500 m alt., caulirrosuletum 1.50 m, envés con indumento dorado blanquecino, hojas jóvenes con indumento blanco amarillento, inflorescencias péndulas, lígulas amarillas, hojas secas cubriendo el tallo, poblaciones abundantes en suelo seco, el ejemplar presenta huellas de quema, 13 May 1968, S. Diaz 42 (holotype: COL; isotype: US!).

**Common names.** Frailejón guacharaco (C), guacharaco (C).

**4.09 *Espeletiopsis insignis*** **(Cuatrec.) Cuatrec.**, Phytologia 35(1): 55. 1976. *Espeletia insignis* Cuatrec., Revista Acad. Colomb. Ci. Exact. 3(11): 432. 1940. **TYPE. COLOMBIA: Norte de Santander:** Hoya del río Chitagá, abajo de Quebrada de Presidente, junto a “Vega Colombia”, bosques 2900–3000 m alt., 21 Jul 1940, J.Cuatrecasas & H.García-Barriga 10071 (holotype: COL; isotypes: BC!, F!, US!).

**Common names.** Frailejón (C), frailejón de Chitagá (C).

**4.10 *Espeletiopsis jimenez-quesadae*** **(Cuatrec.) Cuatrec.**, Phytologia 35(1): 56. 1976. *Espeletia jimenez-quesadae* Cuatrec., Revista Acad. Colomb. Ci. Exact. 3(11): 247. 1940. **TYPE. COLOMBIA: Boyacá:** Cordillera Oriental: Sierra Nevada del Cocuy, hacia La Cueva en La Zanja, Páramo 3700 m alt., 13 Sep 1938, J.Cuatrecasas & H.García–Barriga 1635 (holotype: COL!; isotype: US!).

**Common names.** Frailejón (C), frailejón negro (C).

**4.11 *Espeletiopsis meridensis*** **(Cuatrec.) Cuatrec.**, Phytologia 35(1): 56. 1976. *Espeletia meridensis* Cuatrec., Mutisia 16: 4. 1953. **TYPE. VENEZUELA: Mérida:** Carretera Andina, Páramo de la Negra, 3000 m alt., planta de 0.50 m, tallo floral 1 m, flores amarillas, Nov 1948, H.García–Barriga 13297 (holotype: US!; isotypes: COL, NY, VEN).

**Common names.** Frailejón (V), frailejón de Mérida*.

**Note.** Based on examination of herbarium specimens I suspect that, as with *Espeletiopsis* ×*cristalinensis* (see section of hybrids for *Libanothamnus*), this is another intergeneric hybrid, probably between *Libanothamnus neriifolius* and a species of *Espeletia*. However, more material is needed to confirm this hypothesis.

**4.12 *Espeletiopsis muiska*** **(Cuatrec.) Cuatrec.**, Phytologia 35(1): 56. 1976. *Espeletia muiska* Cuatrec., Revista Acad. Colomb. Ci. Exact. 3(11): 429. 1940. **TYPE. COLOMBIA: Boyacá:** Páramo de Guantiva, Alto de Canutos, vert. sur, 3300 m alt., 3 Aug 1940, J.Cuatrecasas 10359 (holotype: US!; isotype: COL!); additional collections: ibidem: Páramo de Guantiva, Alto de Canutos, 3200 m alt., 17 Jul 1940, J.Cuatrecasas & H.García–Barriga 9748 (COL!, US!); ibidem: Páramo de la Rusia, vert. SE, en Boca del Monte, 3300–3400 m alt., 4 Aug 1940, J.Cuatrecasas 10409 (COL!, US!); ibidem: Páramo de Arcabuco (entre Arcabuco y Tunja), 2950 m alt., 5 Jul 1940, J.Cuatrecasas 10440 (COL!, US!).

**Common names.** Frailejón (C), frailejón de los muiskas*.

**4.13 *Espeletiopsis pannosa*** **(Standl.) Cuatrec.**, Phytologia 35(1): 56. 1976. *Espeletia pannosa* Standl., Amer. J. Bot. 2: 480. 1915. **TYPE. VENEZUELA: Trujillo:** Páramo del Jabón, 3,000–3,200 m, Oct 1910, A.Jahn 165 (holotype: US!).

*Espeletia sericea* Cuatrec., Ciencia (Mexico) 6: 263, fig. 4. 1945. **TYPE. VENEZUELA: Mérida:** Mucurubá, 3500–4000 m alt., n. v. Frailejón chirique, 18 Jul 1930, W.Gehriger 342 (holotype: VEN; isotype: G!).

**Common names.** Frailejón (V), frailejón chirique (V), frailejón plateado (V).

**4.14 *Espeletiopsis petiolata*** **(Cuatrec.) Cuatrec.**, Phytologia 35(1): 56. 1976. *Espeletia petiolata* Cuatrec., Revista Acad. Colomb. Ci. Exact. 4: 338. 1941. **TYPE. COLOMBIA: Santander:** Cordillera Oriental: Páramo del Almorzadero, región media, 3500 m alt.; 20 Jul 1940, J.Cuatrecasas & H.García-Barriga 9973 (holotype: BC!, US!; isotypes: COL!, F!).

*Espeletia petiolata* f. *paniculata* Cuatrec., Revista Acad. Colomb. Ci. Exact. 5: 22. 1942. **TYPE. COLOMBIA: Santander:** Cordillera Oriental: Páramo del Almorzadero, región media, 3500 m alt., 20 Jul 1940, J.Cuatrecasas & H.García-Barriga 9973 (holotype: US!; isotypes: BC!, COL!, F!).

**Common names.** Frailejón (C), frailejón de Almorzadero*.

***Espeletiopsis petiolata* var. *escobensi*s** **(Cuatrec.) M. Diazgranados, comb. nov.** *Espeletia petiolata* var. *escobensis* Cuatrec., Revista Acad. Colomb. Ci. Exact. 5: 22. 1942. **TYPE. COLOMBIA: Boyacá:** Cordillera Oriental, entre Soatá y Cocuy en el Alto de Escobal, páramo 3900 m alt., 15 Sep 1938, J.Cuatrecasas & H.García-Barriga 1760 (holotype: COL!; isotype: US!).

***Espeletiopsis petiolata* f. *corymbos*a** **(Cuatrec.) M. Diazgranados, comb. nov.***Espeletia petiolata* f. *corymbosa* Cuatrec., Revista Acad. Colomb. Ci. Exact. 5: 22. 1942. **TYPE. COLOMBIA: Norte de Santander:** quebrada de Presidente en la alta hoja del río Chitagá, 3100 m alt., panicula valde reducta, corymbum Espeletia corymbosae simulans, folia typo similia sed generaliter angustiora, 28 Nov 1941, J.Cuatrecasas 13479 (holotype: COL!; isotypes: US!, F!).

***Espeletiopsis petiolata* f. *medi*a** **(Cuatrec.) M. Diazgranados, comb. nov.***Espeletia petiolata* f. *media* Cuatrec., Revista Acad. Colomb. Ci. Exact. 5: 22. 1942. **TYPE. COLOMBIA: Santander:** Páramo del Almorzadero, vertiente norte, 3600–3800 m alt., panicula typo minus evoluta, folia minus elongata, ligulae breviores (9 mm), 28 Nov 1931, J.Cuatrecasas 13497 (holotype: COL!; isotypes: US!, F!).

***Espeletiopsis petiolata* f. *reduct*a** **(Cuatrec.) M. Diazgranados, comb. nov.** *Espeletia petiolata* f. *reducta* Cuatrec., Revista Acad. Colomb. Ci. Exact. 4: 338. 1941. **TYPE. COLOMBIA: Santander:** Cordillera oriental, Páramo del Almozadero, region media, 3500–3700 m, acaulirrosuletum, 20 Jul 1940, J.Cuatrecasas 9987-A (lectotype: COL!, here designated).

**4.15 *Espeletiopsis pleiochasia*** **(Cuatrec.) Cuatrec.**, Phytologia 35(1): 56. 1976. *Espeletia pleiochasia* Cuatrec., Revista Acad. Colomb. Ci. Exact. 3: 432. 1940. **TYPE. COLOMBIA: Boyacá:** Quebrada de Becerra, al NW de Duitama, bosques y matorrales, 3000–3100 m alt., 4 Aug 1940, J.Cuatrecasas 10399 (holotype: COL!; isotypes: BC!, COL!, F!, HPUJ!, U, US!).

**Common names.** Frailejón (C), frailejón de Duitama*.

***Espeletiopsis pleiochasia* var. *socotan*a** **(Cuatrec.) M. Diazgranados, comb. nov.** *Espeletia pleiochasia* var. *socotana* Cuatrec., Phytologia 31(4): 328. 1975. **TYPE. COLOMBIA: Boyacá:** Municipio de Socha, subpáramo seco, 2900–3000 m alt, Apr 1973, A.M.Cleef 9870 (holotype: US!; isotypes: COL!, U, US!). Additional collections: ibidem: hoya del rio Socotá (afluente del rio Chicamocha), cerros áridos, rocosos en el flanco izquierdo, arriba de la carretera después del empalme tronco 1 m alto 4 cm diam, cubierto de hoja marcescente, hoja clara, algo ruda haz, más clara envés; jóvenes verde amarillento–blanquecino seríceo, inflorescencias secas excepto algun raro cortas, marcescentes, reflejas, 4 Apr 1973, J.Cuatrecasas & R.Jaramillo 28730 (COL!, US!); id., 2940 m, rósulas y caulirrósulas verdoso–grisáceo claras, tronco hasta 30 cm, involucro verde, lígulas amarillas, hojas distales y cogollo cinéreo blanquecinos, densamente tomentoso–vellosos y algo seríceos, las viejas rudas en la haz con vello mas escaso; barbas de la vaina blanca, 5 Apr 1973, J.Cuatrecasas & R.Jaramillo 28734 (US!); id., 2900 m, ladera seca, caulirrosuleto 1.5 m, hojas con indumento blanco, capítulos 7 mm diam, brácteas invol. verde limón, brácteas ligulíferas verde oscuro, lígulas amarillas, 9 Oct 1971, R.Jaramillo, G.Lozano & S.Díaz 5017 (COL!, US!).

**4.16 *Espeletiopsis pozoensis*** **(Cuatrec.) Cuatrec.**, Phytologia 35(1): 56. 1976. *Espeletia pozoensis* Cuatrec., Ciencia (Mexico) 6: 266. 1945. **TYPE. VENEZUELA: Mérida:** between San José and Beguilla, Páramo de Pozo Negro, 8500–10500 ft, leaves in dense rosette, gray white on both sides; rays pale yellow, disk dull yellow, 3 May 1944, J.Steyermark 56278 (holotype: VEN; isotypes: F!, NY!; photos of VEN and F specimens in US!; photo NY 4564A).

**Common names.** Frailejón (V), frailejón chirique (V), frailejón plateado (V), frailejón plateado chico (V).

**4.17 *Espeletiopsis purpurascens*** **(Cuatrec.) Cuatrec.**, Phytologia 35(1): 56. 1976. *Espeletia purpurascens* Cuatrec., Revista Acad. Colomb. Ci. Exact. 5: 16. 1942. **TYPE. COLOMBIA: Norte de Santander:** Cordillera Oriental, Páramo de Tamá, alrededores de La Cueva, 3000–3200 m alt., entre arbustos en subpáramo, caulirrósula, tallo de 3 m, flores liguladas púrpura, reflejas, flores del disco rojizas, 28 Oct 1941, J.Cuatrecasas, R.E.Schultes & E.Smith 12689 (holotype: COL!; isotypes: BC!, COL!, F!, US!).

**Common names.** Frailejón (C, V), frailejón purpúreo (C, V), frailejón de árbol (C, V).

**Figure 1.**
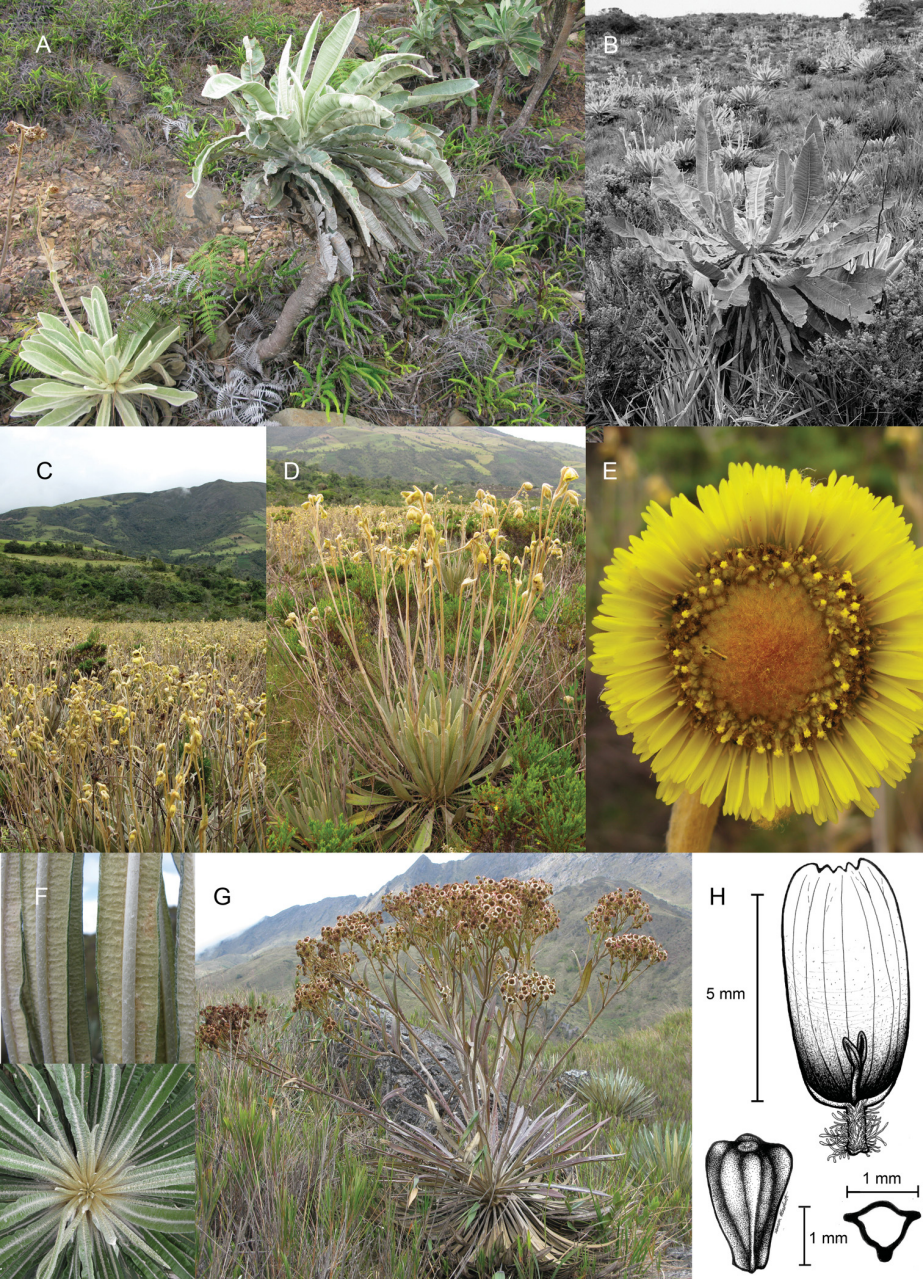
**A–B** *Espeletiopsis* ×*cristalinensis* in the topolocality (**A** coll. M.Diazgranados 4088 (center), growing between *Espeletia aristeguietana* (bottom left corner) and *Libanothamnus neriifolius* (top right corner) **B** coll. J.Cuatrecasas 28994) **C-E** first record of *Espeletia steyermarkii* for Colombia (coll. M.Diazgranados 3862) **F–I** first record of *Ruilopezia cardonae* for Colombia (coll. M.Diazgranados & R.Sánchez 3922). Photographs **A**, **C–G**, **I** by M.Diazgranados, **B** by J.Cuatrecasas; illustrations in **H** by Lauren Merchant.

**Note.** A very dense population of this species was found for the first time in this country, in an azonal (atypical) páramo for the area, on soils of poor drainage. This is the same environment in which the species is usually found in Venezuela. Some individuals had extremely long opposite floral branches starting in the first portion of the scape of the synflorescences, while others plants had alternate branches, which is a characteristic reported for *Espeletia steyermarkii*. A second population in Colombia has been found in another azonal páramo in the area of the Vereda el Azul (Mun. Toledo).

**Figure 2.**
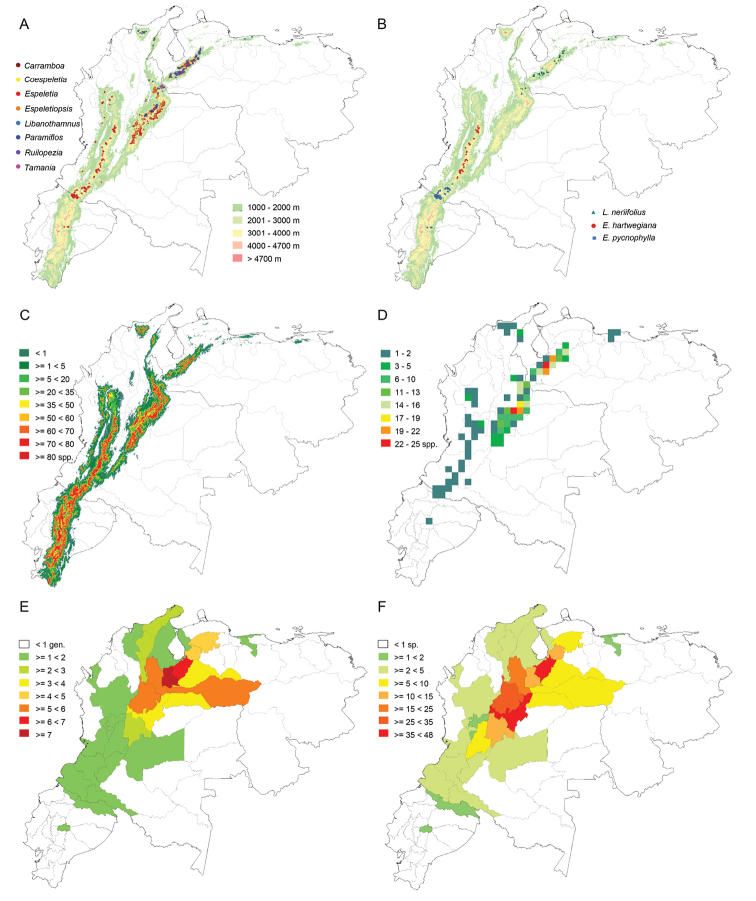
Geographic distribution and species richness of the subtribe *Nomen nudum*. **A** geographic distribution of the genera (colored dots); background color denotes mountains over 1000 m of elevation **B** distribution of three widespread species **C** species richness throughout the elevation, with classes every 200 m **D** species richness by area (squares: 0.4° × 0.4°); **E** richness of genera by state **F** species richness by state.

**4.18 *Espeletiopsis rabanalensis*** **S. Díaz & Rodr.-Cabeza**, Revista Acad. Colomb. Ci. Exact. 32 (125): 456–458 figs 1, 2A–B. 2008. **TYPE. COLOMBIA: Boyacá:** Municipio de Samacá, Páramo del Rabanal, 3412 m de alt,, 3–5 Oct 2007, B.V.Rodr.-Cabeza, f.Márquez 1895 (holotype: COL; isotypes: COL!, UIS).

**Common names.** Frailejón (C), frailejón de Rabanal*.

**4.19 *Espeletiopsis sanchezii*** **S. Díaz & Obando**, Revista Acad. Colomb. Ci. Exact. 28(108): 324, fig. 1. 2004. **TYPE. COLOMBIA: Norte de Santander:** Pamplona, sector de La Lejia, Páramo de Tierranegra, 3200–3300 m L.R.Sánchez, M.A.Murcia & W.Valencia 7281 (holotype: COL; isotype: COL!).

**Common names.** Frailejón (C), frailejón de Tierranegra*.

**4.20 *Espeletiopsis santanderensis*** **(A. C. Sm.) Cuatrec.**, Phytologia 35(1): 56. 1976. *Espeletia santanderensis* A. C. Sm., Brittonia 1(7): 527. 1935. **TYPE. COLOMBIA: Santander:** Páramo de Vetas, alt. 3400–4000 m, 16 Jan 1927, E.P.Killip & A.C.Smith 17422 (holotype: NY!; isotypes: G, NY); additional collections: ibidem: edge of Páramo de Santurbán, near Vetas, E.P.Killip & A. C. Smith 17587 (G, N, Y); ibidem: Páramo de Mogotocoro, near Vetas, E.P.Killip & A.C.Smith 17603 (G, N, Y), 17622 (G, N, Y); ibidem: páramo Rico, near Vetas, E.P.Killip & A.C.Smith 17667 (G, N, Y); ibidem: Páramo de Santurbán, above Tona, E.P.Killip & A.C.Smith 19552 (G, N, Y); Norte de Santander: Páramo Viejo, Purdie (G, K!, P); ibidem: Páramo near Ocaña, L.J.Schlim 332 (P).

**Common names.** Frailejón (C), tache (C).

**4.21 *Espeletiopsis sclerophylla*** **(Cuatrec.) Cuatrec.**, Phytologia 35(1): 56. 1976. *Espeletia sclerophylla* Cuatrec., Revista Acad. Colomb. Ci. Exact. 3: 436. 1940. **TYPE. COLOMBIA: Santander:** Páramo del Almorzadero, extremo sur, Peralonso, 3200 m alt., 19 Jul 1940, J.Cuatrecasas & H.García-Barriga 9929 (holotype: COL!; isotypes: F!, US!).

**Common names.** Frailejón (C), frailejón hojiduro*.

**4.22 *Espeletiopsis trianae*** **(Cuatrec.) Cuatrec.**, Phytologia 35(1): 56. 1976. *Espeletia trianae* Cuatrec., Revista Acad. Colomb. Ci. Exact. 5: 18. 1942. **TYPE. COLOMBIA: Norte de Santander:** páramos de Pamplona, 3000 m alt., árbol, Jun 1851, Triana 2476-5 (holotype: COL!).

**Common names.** Frailejón (C), frailejón de Triana*.

**Hybrids**

***Espeletiopsis ×almorzana* (Cuatrec.) Cuatrec.** **(= *Espeletiopsis petiolata* (Cuatrec.) Cuatrec.** × ***Espeletiopsis sclerophylla* (Cuatrec.) Cuatrec.)**

*Espeletia almorzana* Cuatrec., Revista Acad. Colomb. Ci. Exact. 4(14): 340, figs. 5, 6H. 1941. **TYPE. COLOMBIA: Santander:** Cordillera Oriental: Páramo del Almorzadero, región media 3.500 m alt., 20 Jul 1940, J.Cuatrecasas & H.García-Barriga 9987 (holotype: US!).

*Espeletia almorzana* f. *latifolia* Cuatrec., Revista Acad. Colomb. Ci. Exact. 4(14): 340, figs. 5, 6H. 1941. **TYPE. COLOMBIA: Santander:** Páramo del Almorzadero, vertiente norte, 3600–3800 m alt., 28 Nov 1941, J.Cuatrecasas 13503 (holotype: COL!).

**Common names.** Frailejón (C).

***Espeletiopsis ×bogotensis*** **(Cuatrec.) Cuatrec. (= *Espeletiopsis corymbosa* (Humb. & Bonpl.) Cuatrec.** × ***Espeletia grandiflora* Humb. & Bonpl.)**

*Espeletia bogotensis* Cuatrec., Revista Acad. Colomb. Ci. Exact. 3(11): 427. 1940. **TYPE. COLOMBIA: Cundinamarca:** Macizo de Bogota en Cerro de Monserrate, vert. orient., páramo 3000 m alt, 28 Jan 1940, J.Cuatrecasas 7998 (holotype: COL!; isotypes: COL!, F!, US!); et ibidem: Páramo de Usaquén, 3000 m alt., 20 Jan 1940, J.Cuatrecasas 7995 (paratype: US!); additional collections: ibidem: Páramo de Guasca, 3000–3400 m alt., tallo simple de 1’5 m alt., cubierto por denso estuche de hojas secas persistentes y terminado por un rosetón de hojas lanudas, E.P.Killip 34056 (COL).

**Common names.** Frailejón (C), frailejón de Bogotá*.

**5 *Libanothamnus*** **Ernst**, Vargasia 7: 186. 1870. **TYPE.** *Libanothamnus neriifolius* (Bonpl. & Humb.) Ernst. (= *Trixis neriifolia* Bonpl. & Humb.)

**5.01 *Libanothamnus arboreus*** **(Aristeg.) Cuatrec.**, Phytologia 35(1): 50. 1976. *Espeletia arborea* Aristeg., Bol. Soc. Venez. Ci. Nat. 20(93): 286–287. 1959. **TYPE. VENEZUELA: Trujillo:** Guirigay, near Peña Blanca, 3200 m, Aug 1958, L.Aristeguieta & Medina 3635a (holotype: VEN).

**Common names.** Incienso (V), punta de lanza grande (V).

***Libanothamnus arboreus* var. *lancifoliu*s** **Cuatrec.,** *ined*. (Forthcoming publication in Cuatrecasas in press.)

**5.02 *Libanothamnus banksiaefolius*** **(Sch. Bip. & Ettingsh.) Cuatrec.**, Phytologia 35(1): 50. 1976. *Espeletia banksiaefolia* Sch. Bip. & Ettingsh. ex Wedd., Chlor. And. 1: 67. 1856. **TYPE. VENEZUELA: Mérida:** Sierra Nevada de Mérida, 10000–10500 ft, arbuste fl. blanches, Jun 1847, V.A.Funck & L.J.Schlim 1550 (holotype: P (Preté No. 736); isotypes: G, GH, LE, P).

**Common names.** Frailejón de árbol (V), frailejón punta de lanza grande (V), incienso (V), punta de lanza (V), tabacón (V).

***Libanothamnus banksiaefolius* subsp. *granatesianu*s** **(Cuatrec.) Cuatrec.**, *ined*. (Forthcoming publication in Cuatrecasas in press.)

*Espeletia granatesiana* Cuatrec.

*Libanothamnus granatesianus* (Cuatrec.) Cuatrec.

**Common names.** Frailejón de árbol (V), incienso (V), punta de lanza (V), punta de lanza grande (V), tabacón (V).

**5.03 *Libanothamnus divisoriensis* Cuatrec.**, Phytologia 47(1): 1–3. 1980. **TYPE. VENEZUELA: Zulia:** Sierra de Perijá, boundary with COLOMBIA: 3000 m, flowers dirty cream white, 27 Jun–5 Jul 1974, S.S.Tillett & K.W.Hönig 746–746 (holotype: US!; isotypes: UCV, VEN).

**Common names.** Incienso (V), tabaquillo de la frontera (C, V).

**5.04 *Libanothamnus griffinii*** **(Ruiz-Terán & López-Fig.) Cuatrec.**, Phytologia 35(1): 50. 1976. *Espeletia griffinii* Ruiz-Terán & López-Fig., Rev. Fac. Farm. Univ. Andes Mérida 17: 7–13, figs. 3–6. 1976. **TYPE. VENEZUELA: Trujillo:** Páramo de Guaramacal, Boconó, 2600 m, 3 Aug 1975, L.Ruiz-Terán, M.López-Figueiras & D.Griffin 12606 (holotype: MERF!; isotype: US!).

**Common names.** Incienso (V), incienso de Guaramacal*.

**5.05 *Libanothamnus liscanoanus*** **(Cuatrec.) Cuatrec.**, Phytologia 35(1): 51. 1976. *Espeletia liscanoana* Cuatrec., Phytologia 27(1): 41–44. 1973. **TYPE. VENEZUELA: Lara:** Páramo del Jabón, 3100–3200 m, tree 8–10 m, 20–25 cm diameter, 2 Nov 1969, J.Cuatrecasas, L.Ruiz-Terán & M.López-Figueiras 28206 (holotype: US!; isotype: MERF!).

**Common names.** Incienso (V), incienso de Carache*.

**5.06 *Libanothamnus lucidus*** **(Aristeg.) Cuatrec.**, Phytologia 35(1): 51. 1976. *Espeletia lucida* Aristeg., Fl. Venez. 10(1): 420–421. 1964. **TYPE. VENEZUELA: Mérida:** without collector and exact locality, 3800 m, ULA-1001 (holotype: VEN); ibidem: camino al Pico Bolívar, 9 Apr 1951, Uscátegui 1001 (isotype: MER).

**Common names.** Frailejón de madera (V), incienso (V).

**5.07 *Libanothamnus neriifolius*** **(Bonpl. ex Humb.) Ernst**, Vargasia 7: 186. 1870. **TYPE. COLOMBIA:** Moritz 372 (B). *Baillieria nereifolia* (Kunth) Bonpl. ex Kunth, Nov. Gen. Sp. Pl. 4: 289. 1820; Folio. 4: 227. 1818. *Clibadium neriifolium* (Bonpl. ex Kunth) DC., Prodr. Syst. Nat. Reg. Veg. 5: 507. 1836. *Espeletia neriifolia* (H.B.K.) Sch. Bip. ex Wedd., Chlor. And. 1: 67-68. 1856. **TYPE. COLOMBIA: Norte de Santander:** Páramo de Tamá, Hoya de Samaria, 2600–2900 m, rays white, 29 Oct 1941, J.Cuatrecasas, R.E.Schultes & E.Smith 12721 (holotype: COL!; isotypes: BC!, COL!, F!, GH, HPUJ!, US!).

*Trixis neriifolia* Bonpl. ex Humb., Voy. Reg. Equin. Rel. 1: 605. 1814. As T. nereifolia, typographic error. **TYPE. VENEZUELA:** Syngenesia necessaria, Trixis neriifolia foliis lanceolatis integerrimis subtus laniculatis, habitat in Caracas, incienso, Silla de Caracas, Humboldt s.n. (Willdenow 16672 lectotype: B, photo 16103: F); ibidem: Caracas, incienso, Buphthalmum, Silla de Caracas, in other label “Andromachia? Trixis neriifolia Willd! Espeletia neriifolia Sch. Bip. 19/10/55, Silla de Caracas, Herbier donné par M.Bonpland in 1833, Jan 1800, No. 652, (isolectotype: P).

**Common names.** Frailejón de árbol (C, V), frailejón de resina (V), frailejón resino (V), incienso (C, V), oreja de burro (C), resino (V), tabaquillo trementino (C), trementino (C).

***Libanothamnus neriifolius* var. *boconensi*s** **Cuatrec.**, Phytologia 47(1): 6. 1980. **TYPE. VENEZUELA: Trujillo:** Páramo de La Cristalina, 2300 m, tree 6–10 m, rays white, 30 Oct 1969, J.Cuatrecasas, L.Ruiz-Terán & M.López-Figueiras 28190 (holotype: US!; isotype: MERF!).

**Common names.** Incienso (V), incienso de Boconó*.

***Libanothamnus neriifolius* var. *columbicu*s** **(Cuatrec.) Cuatrec.**, *ined*. (Forthcoming publication in Cuatrecasas in press.)

*Espeletia neriifolia* var. *columbicus* Cuatrec.

**Common names.** Incienso (C), incienso colombiano*, oreja de burro (C), tabaquillo trementino (C), trementino (C).

***Libanothamnus neriifolius* var. *cristamontis* Cuatrec.**, *ined*. (Forthcoming publication in Cuatrecasas in press.)

*Libanothamnus cristamontis* Cuatrec.

**Common names.** Incienso (V), incienso de Dinira*.

***Libanothamnus neriifolius* var. *turmalensi*s** **Cuatrec.**, Phytologia 47(1): 7. 1980. **TYPE. VENEZUELA: Trujillo:** Páramo del Turmal, east of Carache, 2800–2900 m, tree 5 m, rays creamy–white, 3 Nov 1969, J.Cuatrecasas, L.Ruiz-Terán & M.López-Figueiras 28239 (holotype: US!; isotype: MERF!).

**Common names.** Incienso (V), incienso del Turmal*.

**5.08 *Libanothamnus occultus*** **(S. F. Blake) Cuatrec.**, Phytologia 35(1): 51. 1976.

*Espeletia occulta* S. F. Blake, Contrib. U.S. Nat. Herb. 20: 537. 1924. **TYPE. VENEZUELA: Mérida:** Páramo de Quirorá, 3000 m, 8 Oct 1921, Jahn 730 (holotype: US!; isotype: MERF!).

*Libanothamnus occultus* var. *salomonii* Cuatrec. & López-Fig., Phytologia 61(1): 51–53. 1986. **TYPE. VENEZUELA: Táchira:** Pico de Horma, 7.5 Km SE of Mesa de Quintero, 3100 m, western slopes, subpáramo, tree up to 5 m, ligules pale–yellowish, leaves tawny beneath, Táchira, VENEZUELA, 11 Jan 1985, M.López-Figueiras, Rodríguez & Rengifo 31344 (holotype: US!; isotypes: F!, HPUJ!, MERF!, NY!).

**Common names.** Incienso (V), tabaquillo (C), tabaquillo oscuro*.

***Libanothamnus occultus* subsp. *glossophyllu*s** **(Mattf.) Cuatrec.**, *ined*. (Forthcoming publication in Cuatrecasas in press.)

*Espeletia glossophylla* Mattf.

*Espeletia subneriifolia* Cuatrec.

*Libanothamnus glossophyllus* (Mattf.) Cuatrec.

*Libanothamnus subneriifolius* (Cuatrec.) Cuatrec.

**Common names.** Frailejón (C), incienso (C), nabalda (Kogi), tabaco de la sierra*.

***Libanothamnus occultus* subsp. *humberti*i** **(Cuatrec.) Cuatrec.**, *ined*. (Forthcoming publication in Cuatrecasas in press.)

*Espeletia humbertii* Cuatrec.

*Libanothamnus humbertii* (Cuatrec.) Cuatrec.

**Common names.** Frailejón de árbol (V), frailejonote (V), incienso (V), punta de lanza (V), punta de lanza amarillo (V), punta de lanza pequeño (V), tabacote (V).

***Libanothamnus occultus* subsp. *oroquensi*s** **Cuatrec.**, *ined*. (Forthcoming publication in Cuatrecasas in press.)

**Common names.** Incienso (C), tabaquillo de Oroque (C).

**5.09 *Libanothamnus parvulus*** **Cuatrec.**, Phytologia 47(1): 3–4. 1980. **TYPE. VENEZUELA: Lara:** Páramo de Cendé, 2900 m at Laja del Díctamo, short tree, 10 Jun 1971, L.Ruiz-Terán & M.López-Figueiras 2036 (holotype: US!; isotype: MERF!).

**Common names.** Incienso (V), incienso hojipequeño*.

**5.10 *Libanothamnus spectabilis*** **(Cuatrec.) Cuatrec.**, Phytologia 35(1): 51. 1976. *Espeletia spectabilis* Cuatrec., Phytologia 27(1): 46–49. 1973. **TYPE. VENEZUELA: Mérida:** Zanjón del Cupís, in Páramo de San Jóse, Andean silva, 3100 m, caulirosula 8–10 m, 18–21 Nov 1972, M.López-Figueiras, H.A.Rodríguez & J.&M.Wurdack 8912 (holotype: US!; isotypes: BC!, F!, G!, HPUJ!, K!, MERF!, NY!, VEN!).

**Common names.** Frailejón de monte (V), incienso (V).

**5.11 *Libanothamnus tamanus*** **(Cuatrec.) Cuatrec.**, Phytologia 35(1): 51. 1976. *Espeletia tamana* Cuatrec., Phytologia 27(3): 171–173. 1973. **TYPE. VENEZUELA: Táchira:** Páramo de Tamá, NW end, Quebrada del Reposo, timberline, 2800 m, tree 4 m, white ligules, 2 Jun 1973, L.Ruiz-Terán & M.López-Figueiras 8915 (holotype: US!; isotype: MERF!).

**Common names.** Incienso (C, V), tabaquillo de Tamá*.

**Hybrids**

***Espeletiopsis ×cristalinensis*** (**Cuatrec.) Cuatrec. (= *Libanothamnus neriifolius* (Bonpl. ex Humb.) Ernst** × Espeletia aristeguietana **Cuatrec.)**, Phytologia 35(1): 55. 1976.

*Espeletia cristalinensis* Cuatrec., Phytologia 27(1): 169. 1973. **TYPE. VENEZUELA: Trujillo:** Distrito de Boconó, Páramo de la Cristalina, 2500–2600 m alt., en lomas semi clareadas de monte andino, porte gris verdoso claro o ceniciento verdoso, cogollo blanquecino, tronco erecto hasta 60 cm, 7 cm de diám., cubierto de hoja marcescente, simple o ramoso sobre la base con varios grandes rosetones, 8–12 sinflorescencias axilares robustas, hojas coriáceas flexibles o rígidas, verdoso grisáceas haz, claras envés, vainas semiamplectantes, lígulas amarillo vivo, flósculos amarillos, círculo ligular 30 mm, disco 13 mm, 17 Feb 1973, J.Cuatrecasas, L.Ruiz-Terán & M.López-Figueiras 28556 (holotype: US!; isotype: MERF).

**Common names.** Frailejón (V), incienso cenizo de la Cristalina*.

**Note.** Both molecular and morphological evidence suggest the hybrid nature of *Espeletiopsis ×cristalinensis*. Based on the author’s field observations in the topolocality, only a few sparse individuals were found in the contact zone of two large populations of the species suggested in the hybrid formula (Fig. 1A). *Espeletiopsis* ×*cristalinensis* has characteristics of both *Libanothamnus* and *Espeletia*, which probably confused Cuatrecasas, who was never convinced about the position of this form, and had placed it within *Libanothamnus* in his notes. Leaves with open sheaths and abundant hairs are more similar to *Espeletia* species, while numerous characteristics from *Libanothamnus* are expressed in the synflorescences. A photograph at US of the live paratype (L.Ruiz-Terán & M.López-Figueiras 2257) shows the plant growing between *Libanothamnus neriifolius* plants, and having leaves with undulate margin and unhealthy appereance, which I have seen as frequent characters in other hybrids of the group. Another photograph taken by Cuatrecasas in the topolocality (photo I-4976, coll. J.Cuatrecasas 28994) shows an individual of *Espeletiopsis* ×*cristalinensis* growing isolated in a population of *Espeletiopsis aristeguietana* (Fig. 1B).

***Libanothamnus ×gritaensis*** **Cuatrec. (= *Libanothamnus neriifolius* var. *columbicus* Cuatrec.** × L. occultus **(S. F. Blake) Cuatrec.)**, Phytologia 47(1): 7 (–8). 1980. **TYPE. VENEZUELA: Tachira:** Llano de Campoalegre, cercanías de La Grita hacia Páramo del Batallón, 2500 m, árbol 5 m, hoja coriácea, crasiúscula, verde amarillenta haz, verde cenicienta envés, pedúnculos verde claros, lígulas blancas (blanco crema), photos K 2705–2712, I, 2 Oct 1969, J.Cuatrecasas, M.López-Figueiras, L.Marcano-Berti 27999 (holotype: US!; isotypes: US!, MERF).

**6 *Paramiflos*** **Cuatrec.**, Proc. Biol. Soc. Wash. 108: 748–750. 1995. **TYPE.** *Espeletia glandulosa* Cuatrec. (= *Paramiflos glandulosus* (Cuatrec.) Cuatrec.)

**6.01 *Paramiflos glandulosus*** **(Cuatrec.) Cuatrec.**, Proc. Biol. Soc. Wash. 108: 749. 1995. *Espeletia glandulosa* Cuatrec., Revista Acad. Colomb. Ci. Exact. 3(11): 434, figs. 17, 18, 23, pl. III. 1940. **TYPE. COLOMBIA: Boyacá:** Alto de Canutos, Páramo de Guantiva, southern slope, 3200–3400 m, 3 Aug 1940, J.Cuatrecasas 10360 (holotype: COL; isotypes: COL!, F!, P, US!); additional collections: ibidem: Páramo de Arcabuco, 2950 m alt., 5 Aug 1940, J.Cuatrecasas 10444 (COL, US).

*Espeletia glandulosa* var. *scaberrima* Cuatrec., Brittonia 8(3): 185. 1956. **TYPE. COLOMBIA: Norte de Santander:** Andes de Pamplona, 2800 m, Jun 1851, Triana 1327 (holotype: P; isotypes: P, COL).

*Espeletiopsis glandulosa* (Cuatrec.) Cuatrec., Phytologia 35(1): 55. 1976.

**Common names.** Frailejón (C), frailejón glanduloso (C).

**7 *Ruilopezia*** **Cuatrec.**, Phytologia 35(1): 51, fig. 2. 1976. **TYPE.** *Ruilopezia figueirasii* (Cuatrec.) Cuatrec.

**7.01 *Ruilopezia atropurpurea*** **(A. C. Sm.) Cuatrec.**, Phytologia 35(1): 52. 1936. *Espeletia atropurpurea* A. C. Sm., Brittonia 1(7): 508. 1935. **TYPE. VENEZUELA: Mérida:** Páramo de Quirorá, 3200 m, 8 Oct 1971, Jahn 731 (holotype: US!; isotypes: F!, G, GH, NY!, VEN).

**Common names.** Frailejón (V), frailejón tostado (V).

**7.02 *Ruilopezia bracteosa*** **(Standl.) Cuatrec.**, Phytologia 35(1): 52. 1976. *Espeletia bracteosa* Standl., Amer. J. Bot. 2: 484. 1915. **TYPE. VENEZUELA: Trujillo:** Páramo de la Cristalina, between Boconó and Trujillo, 2900 m, 20 Dec 1910, Jahn 156 (holotype: US!; isotype: VEN).

*Espeletia frailejonota* Aristeg., Bot. Soc. Venez. Ci. Nat. 20(93): 284–285. 1959. **TYPE. VENEZUELA: Trujillo:** Sierra de Guirigay near the Laguna Parida, 3400 m, Aug 1958, L.Aristeguieta & Medina 3576 (holotype: VEN; isotypes: NY!, US!).

*Ruilopezia frailejonota* (Aristeg.) Cuatrec., Phytologia 35(1): 52. 1976.

**Common names.** Frailejón (V), frailejón de potrero*.

**7.03 *Ruilopezia bromelioides*** **(Cuatrec.) Cuatrec.**, Phytologia 35(1): 52. 1976. *Espeletia bromelioides* Cuatrec., Phytologia 29(5): 369–372. 1975. **TYPE. VENEZUELA: Mérida:** Páramo de Los Colorados, potreros de San Rafael, 2600–2700 m, 18–20 Jul 1974, M.López Figueiras & H.A.Rodríguez 9054 (holotype: US!; isotype: MERF!).

**Common names.** Frailejón (V), frailejón bromelia*.

**7.04 *Ruilopezia cardonae*** **(Cuatrec.) Cuatrec.**, Phytologia 35(1): 52. 1976. *Espeletia cardonae* Cuatrec., Revista Acad. Col. Ci. Exact. 5(17): 20–21, fig. 8. 1942. **TYPE. VENEZUELA: Táchira:** Cordillera de Los Andes, Páramo de Tamá, headwaters of Río Oirá, 3100–3300 m, Jul 1939, Cardona 304 (holotype: VEN; isotypes: F!, US!).

**Common names.** Frailejón (V), frailejón de cardo*.

**7.05 *Ruilopezia coloradarum*** **(Cuatrec.) Cuatrec.**, Phytologia 35(1): 52. 1976. *Espeletia coloradarum* Cuatrec., Phytologia 29(5): 372–374. 1975. **TYPE. VENEZUELA: Mérida:** potrero de San Rafael in Páramo de Las Coloradas, 2700 m, 3 Jul 1974, M.López-Figueiras & M.Keogh 9108 (holotype: US!; isotypes: F!, MERF!, NY!).

**Common names.** Frailejón (V), frailejón de las Coloradas*.

**7.06 *Ruilopezia cuatrecasasii*** **(Ruiz-Terán & López-Fig.) Cuatrec.**, Phytologia 35(1): 52. 1976. *Espeletia cuatrecasasii* Ruiz-Terán & López-Fig., Rev. Fac. Farm. Univ. Andes Mérida 14: 5–13. 1974. **TYPE. VENEZUELA: Mérida:** between El Morro & Aricagua, at the border of Laguna Tapada, 2630 m, 9 Jun 1973, L.Ruiz-Terán & M.López-Figueiras 8738 (holotype: MERF!; isotype: US!).

**Common names.** Frailejón (V), frailejón de Cuatrecasas*.

**7.07 *Ruilopezia emmanuelis*** **Cuatrec.**, Phytologia 61(1): 56–58. 1986. **TYPE. VENEZUELA: Trujillo:** Páramo de Las Rosas, between Las Lajas and Barro Amarillo, 2900–3000 m, 8 Mar 1985, M.López-Figueiras & D.Griffin 32405 (holotype: US!; isotypes: F!, G!, K!, MERF!, NY!, US!, VEN!).

**Common names.** Frailejón (V), frailejón de Las Rosas*.

**7.08 *Ruilopezia figueirasii*** **(Cuatrec.) Cuatrec.**, Phytologia 35(1): 52. 1976. *Espeletia figueirasii* Cuatrec., Phytologia 20(8): 475–476. 1971. **TYPE. VENEZUELA: Mérida:** Páramo de Los Granates, in Loma de Paja, 3240 m, 11 Oct 1969, J.Cuatrecasas, L.Ruiz-Terán & M.López-Figueiras 28068 (holotype: US!; isotype: MERF!).

**Common names.** Frailejón guarura (V), frailejón maguey (V), frailejón negro (V).

**7.09 *Ruilopezia floccosa*** **(Standl.) Cuatrec.**, Phytologia 35(1): 52. 1976. *Espeletia floccosa* Standl., Amer. J. Bot. 2: 481. 1915. **TYPE. VENEZUELA: Mérida:** Páramo de Timotes, 3300–4000 m, frailejón plateado, Oct 1910, Jahn 154 (holotype: US!).

**Common names.** Frailejón (V), frailejón brillante (V), frailejón muñeco (V), frailejón niño (V), frailejón plateado (V).

**7.10 *Ruilopezia grisea*** **(Standl.) Cuatrec.**, Phytologia 35(1): 52. 1976. *Espeletia grisea* Standl., Amer. J. Bot. 2: 477–478. 1915. **TYPE. VENEZUELA: Mérida:** Sierra Nevada de Mérida, southern slopes, Chorro Blanco, 3800 m, Jan 1911, Jahn 157 (holotype: US!).

**Common names.** Frailejón (V), frailejón gris (V).

**7.11 *Ruilopezia hanburiana*** **(Cuatrec.) Cuatrec.**, Phytologia 35(1): 52. 1976. *Espeletia hanburiana* Cuatrec., Soc. Venez. Cienc. Nat. 17(85): 86–88, figs. s.n. 1956. **TYPE. VENEZUELA: Mérida:** Páramo de Acequias, 3500 m, 18 Oct 1938, Hanbury-Tracy 130 (holotype: K!; isotypes: NY!, US!).

**Common names.** Frailejón (V), frailejón de Campo Elías*.

**7.12 *Ruilopezia jabonensis*** **(Cuatrec.) Cuatrec.**, Phytologia 35(1): 52. 1976. *Espeletia jabonensis* Cuatrec., Phytologia 23(4): 360–362. 1972. **TYPE. VENEZUELA: Trujillo–Lara:** Páramo del Turmal, Tres Pozos, close to border with state of Lara and Páramo del Jabón, 2800–2850 m, 8 Jun 1971, L.Ruiz-Terán & M.López-Figueiras 1995 (holotype: US!; isotype: MERF!).

**Common names.** Frailejón blanco (V), frailejón plateado (V), frailejón plateado de hoja fina*.

**7.13 *Ruilopezia jahnii*** **(Standl.) Cuatrec.**, Phytologia 35(1): 53. 1976. *Espeletia jahnii* Standl., Amer. J. Bot. 2: 479–480, fig. 5. 1915. **TYPE. VENEZUELA: Táchira:** Páramo del Batallón, 3000 m, Mar 1911, Jahn 155 (holotype: US!; isotype: VEN).

**Common names.** Frailejón (V), frailejón de Táchira*.

**7.14 *Ruilopezia joséphensis*** **(Cuatrec.) Cuatrec.**, Phytologia 35(1): 52. 1976. *Espeletia joséphensis* Cuatrec., Phytologia 29(5): 374–377. 1975. **TYPE. VENEZUELA: Mérida:** Páramo de San José de Acequias, 2600 m, 18–20 Jul 1974, M.López-Figueiras & H.A.Rodríguez 9073 (holotype: US!; isotype: MERF!).

**Common names.** Frailejón (V), frailejón de Don José*.

**7.15 *Ruilopezia leucactina*** **(Cuatrec.) Cuatrec.**, Phytologia 35(1): 52. 1976. *Espeletia leucactina* Cuatrec., Phytologia 29(5): 377–379. 1975. **TYPE. VENEZUELA: Táchira:** Páramo del Batallón, 3000 m, 13 Aug 1974, M.López-Figueiras 9151 (holotype: US!; isotypes: F!, MERF!).

**Common names.** Frailejón (V), frailejón blanco del Batallón*.

**7.16 *Ruilopezia lindenii*** **(Sch. Bip. ex Wedd.) Cuatrec.**, Phytologia 35(1): 53. 1976. *Espeletia lindenii* Sch. Bip. ex Wedd., Chlor. And. 1: 66–67. 1856. **TYPE. VENEZUELA: Mérida:** Páramo del Tambor, 11000–12000 ft, Jul 1843, J.J.Linden 1414 (holotype: P; isotypes: BR, F!, FI, G, K!, LE, P, W).

**Common names.** Frailejón (V), frailejón macho (V), frailejón morado (V).

**7.17 *Ruilopezia lopez-palacii*** **(Ruiz-Terán & López-Fig.) Cuatrec.**, Phytologia 35(1): 53. 1976. *Espeletia lopez-palacii* Ruiz-Terán & López-Fig., Rev. Fac. Farm. Univ. Andes Mérida 17: 13–19, figs. 31–34. 1976. **TYPE. VENEZUELA: Trujillo:** Páramo de Guaramacal, 15 km East of Boconó, 2600 m, 9 Aug 1975, L.Ruiz-Terán, M.López-Figueiras & D.Griffin 12619 (holotype: MERF!; isotype: US!).

**Common names.** Frailejón (V), frailejón piña de Guaramacal*.

**7.18 *Ruilopezia marcescens*** **(S. F. Blake) Cuatrec.**, Phytologia 35(1): 53. 1976. *Espeletia marcescens* S. F. Blake, Contrib. U.S. Nat. Herb. 20: 536–537. 1924. **TYPE. VENEZUELA: Mérida:** Páramo de Quirorá, 2950 m, 24 Feb 1922, Jahn 875 (holotype: US!; isotype: VEN).

**Common names.** Frailejón (V), frailejón marcescente (V).

**7.19 *Ruilopezia margarita*** **(Cuatrec.) Cuatrec.**, Phytologia 35(1): 53. 1976. *Espeletia margarita* Cuatrec., Phytologia 27(1): 49–51. 1973. **TYPE. VENEZUELA: Mérida:** Páramo de Los Granates near Laguna Brava, more or less East of Sierra Nevada de Santo Domingo, near Laguna Brava, 3300 m, 20 May 1971, M.López-Figueiras 8720 (holotype: US!; isotypes: BC!, F!, HPUJ!, K!, MERF!, NY!, U!, VEN!).

**Common names.** Frailejón (V), frailejón perla (V).

**7.20 *Ruilopezia paltonioides*** **(Standl.) Cuatrec.**, Phytologia 35(1): 53. 1976. *Espeletia paltonioides* Standl., Amer. J. Bot. 2: 482–483. 1915. **TYPE. VENEZUELA: Trujillo:** Páramo de las Rosas, 3200 m, Oct 1912, Jahn 159 (holotype: US!; isotype: VEN).

**Common names.** Frailejón (V), frailejón piña (V), frailejón piñón (V).

**7.21 *Ruilopezia ruizii*** **(Cuatrec.) Cuatrec.**, Phytologia 35(1): 53. 1976. *Espeletia ruizii* Cuatrec., Phytologia 23(4): 362–363. 1972. **TYPE. VENEZUELA: Mérida:** Loma de La Libertad in Páramo de Las Coloradas, about 500 m from El Portachuelo (El Ramal) between Santa Cruz de Mora and El Molino, 2750–2800 m, 16 Jan 1971, L.Ruiz-Terán & M.López-Figueiras 1457 (holotype: US!; isotypes: F!, MERF!, NY!).

**Common names.** Frailejón (V), frailejón lanudo (V).

**7.22 *Ruilopezia usubillagae*** **Cuatrec.**, Phytologia 61(1): 53–55. 1986. **TYPE. VENEZUELA: Mérida:** Páramo de Aricagua, 3000 m, 3 Nov 1922, Jahn 1021 (holotype: US!; isotype: VEN).

**Common names.** Frailejón (V), frailejón de Aricagua*.

**7.23 *Ruilopezia vergarae*** **Cuatrec. & López-Fig.**, Phytologia 61(1): 58–61. 1986. **TYPE. VENEZUELA: Trujillo:** Sierra de Barbacoas, La Palma, 2390 m, 4 Apr 1976, M.López-Figueiras 12960 (holotype: US!; isotypes: BC!, F!, K!, MA!, MERF!, NY!, U!, VEN!).

**Common names.** Frailejón (V), frailejón blanco (V), frailejón plateado (V), frailejón plateado hojiancho (V).

**7.24 *Ruilopezia viridis*** **(Aristeg.) Cuatrec.**, Phytologia 35(1): 53. 1976. *Espeletia viridis* Aristeg., Bol. Soc. Venez. Ci. Nat. 20(93): 278–281, figs. s.n. 1959. **TYPE. VENEZUELA: Trujillo:** Sierra de Guirigay, towards Laguna Parida, 3500 m, Aug 1958, L.Aristeguieta & Medina 3570 (holotype: VEN; isotypes: NY!, US!, VEN).

**Common names.** Frailejón (V), frailejón morado (V), frailejón piñón (V).

**Hybrids**

***Ruilopezia jabonensis* (Cuatrec.) Cuatrec.** × R. vergarae **Cuatrec. & López-Fig. VENEZUELA: Trujillo:** Quebrada del Río Turmal, upwards to Páramo del Jabón, subpáramo zone, 3000 m, rosette sessile, leaves shiny sericeous, ray and disc corollas yellow, chromosome no. n = ca. 19, “frailejón plateado”, 3 Nov 1969, J.Cuatrecasas, L.Ruiz-Terán & M.López-Figueiras 28237 (MERF!, US!).

***Ruilopezia vergarae* Cuatrec. & López-Fig. *× R. jabonensis* (Cuatrec.) Cuatrec.** **VENEZUELA: Trujillo:** Páramo del Turmal, Tres Pozos, 2800–2850 m, acaulirosula, leaves silvery-sericeous, sterile, 8 Jun 1971, L.Ruiz-Terán & M.López-Figueiras 1996 (MERF!, US!); Id., Tres Pozos, 2850–2800 m, acaulirosula, leaves 1 cm latitude, sericeous but less shiny than 28541, incipient inflorescence, 21 cm long, 5.5 cm diam. at the middle, seldom at the ecotonical zone between communities of R. vergarae and R. jabonensis, “frailejón plateado hojiancho”, 15 Feb 1973, J.Cuatrecasas, L.Ruiz-Terán & M.López-Figueiras 28545 (MERF!, US!).

**8 *Tamania*** **Cuatrec.**, Phytologia 35(1): 53. 1976. **TYPE.** *Espeletia chardonii* A.C. Sm. (= *Tamania chardonii* Cuatrec.)

**8.01 *Tamania chardonii*** **(A. C. Sm.) Cuatrec.**, Phytologia 35(1): 53–54. 1976. *Espeletia chardonii* A. C. Sm., Bol. Soc. Venez. Ci. Nat. 7(50): 237–238. 1942. **TYPE. VENEZUELA: Táchira:** Páramo de Tamá, El Paramito, 2550 m, Frontera Colombo-Venezolana, 25 Aug 1939, A.Escalona & Chardon 78 (holotype: US!; isotypes: NY!, VEN, fragm. isotype: COL; photo 4555A, 4556A: NY!, BG).

*Espeletia leporina* Cuatrec., Revista Acad. Colomb. Ci. Exact. 5(17): 17–18, figs. 3–5; 9C, D; pl. I. 1942. **TYPE. COLOMBIA: Norte de Santander:** Alto del Venado, between Samaria and Toledo, in forest 2300–2400 m, 31 Oct 1941, J.Cuatrecasas, R.E.Schultes & E.Smith 12813 (holotype: COL!; isotypes: A, COL!, F!, HPUJ!, US!).

**Common names.** Frailejón (V), frailejón grande (V), tabaquero (C, V).

**Excluded names**

Some herbarium specimens hold names that have never been published: *Coespeletia aurantia* (Aristeg.) Cuatrec., *Espeletia brassicoidea* var. *lancifolia* Cuatrec., *Espeletia conglomerata* var. *lanceolata* Cuatrec., *Espeletia cristalina* Cuatrec., *Espeletia rosea* Cuatrec., *Espeletia tunjana* f. *magnificens* Cuatrec. and *Espeletia* ×*haughtii* Cuatrec.

Other excluded names are:

***Espeletia amplexicaulis* Nutt.**, J. Acad. Nat. Sci. Philadelphia 7: 38. 1834 (= *Wyethia amplexicaulis* (Nutt.) Nutt.).

***Espeletia helianthoides* Nutt.**, J. Acad. Nat. Sci. Philadelphia 7: 39. 1834 (= *Balsamorhiza sagittata* (Pursh) Nutt.).

***Espeletia sagittata* Nutt.**, J. Acad. Nat. Sci. Philadelphia 7: 39. 1834 (= *Balsamorhiza sagittata* (Pursh) Nutt.).

***Tamananthus crinitus* V.M.Badillo**, Ernstia 30: 25–28. 1985. Explanation follows.

**Exclusion of *Tamananthus* V.M.Badillo**

The inclusion of the monotypic genus *Tamananthus* V.M.Badillo within the subtribe *Espeletiinae* was proposed by Panero (2007). There is only one collection for the described species in the genus:

***Tamananthus crinitus* V.M.Badillo**, Ernstia 30: 25–28. 1985, and Ernstia (2 Etapa) 1–2(2): 17–19. 1992. **TYPE. VENEZUELA: Táchira:** distrito Junín, hacia el extreme nor-occidental del gran Páramo de Tamá, cabecera semi-boscosa de la quebrada El Reposo, unos 6–7 km al norte de Villa Páez, 2800 m, hierba o arbusto, ramitas rojo–purpúra, involucro verde intense con zonas rojo intense, ligulas amarillo-intenso, L.Ruiz-Terán & M.López-Figueiras 8930 (holotype: MERF; isotypes: MY, US!).

The specimen was poorly preserve and glued to the herbarium sheet, and does not allow a comprehensive examination. The following characteristics do not fit within the synapomorphies of the *Nomen nudum*: herbaceous or shrubby habit, alternate leaves, bilabiate corolla with the 2-lobed-lip well developed, bidentate style, 7–8 ray flowers in total, isomorphic uncompressed cypselae with prominent carpopodium, and fertile disk flowers. Some species (*Coespeletia* spp. and a few *Espeletia* spp.) in *Nomen nudum* show bilabiate ray flowers, sometimes with one or two lobes, although this seems to be an inconsistent character within the species. However, the lip in *Nomen nudum* species, when present, is never as developed as in *Tamananthus crinitus*, and lobes in *Nomen nudum* are in general shorter, deeper and more divergent from each other. The morphological characteristics in the specimen and in the description do not conclusively place this taxon with the *Nomen nudum*. DNA amplifications have not been successful until now, but AFLP data suggest that this species is closer to *Ichthyothere* Mart. and *Smallanthus* Mack. than to the *Nomen nudum*. I propose excluding this genus from the subtribe until more material can be studied morphologically and genetically.

### Frequency of hybrids in the subtribe *Nomen nudum*

A total of 27 hybrids are included here. From the 4408 herbarium specimens examined, 122 (2.8%) were hybrids. However these numbers do not represent the frequency of hybridization events. It is unquestionable that hybridization is an important process in the subtribe. During my field observations, I was able to collect and photograph dozens of putative hybrids, which occur typically in contact zones of two parapatric populations (e.g. between the usually low-elevation populations of *Libanothamnus* and the high-elevation populations of *Espeletia*), or in areas where sympatric species have large populations (e.g. páramos with *Espeletia grandiflora* and *Espeletia argentea*, or with *Espeletia schultzii* and *Espeletia weddellii*). However, the frequency of hybridization is overemphasized in the botanical collections, because hybrids are the “different ones” and are usually more often collected by inexpert collectors who believe they are novelties to science.

It is interesting to note that hybridization appears to be more frequent at both extremes of the altitudinal range. In the superpáramos it is relatively frequent between *Coespeletia* species and other sympatric taxa of the subtribe (e.g. *Espeletia schultzii*). In the subpáramos it is common between *Carramboa*, *Espeletiopsis*, lower-elevation *Espeletia* (e.g. *Espeletiopsis aristeguietana*, *Espeletiopsis argentea*, *Espeletiopsis boyacensis*, etc.), *Libanothamnus* and lower-elevation *Ruilopezia* species (e.g. *Ruilopezia marcescens*, *Ruilopezia jabonensis*, *Ruilopezia vergarae*, etc.).

Even more intriguing is that when hybridization in these altitudinal extremes occurs, the synflorescence of the hybrids are often more similar to the species that is adapted to lower elevations, while the leaves are more similar to the species adapted to higher elevations. For instance, *Espeletiopsis* ×*cristalinensis* is found in the upper range of *Libanothamnus neriifolius* and the lower range of *Espeletia aristeguietana*. It has very pubescent thick leaves like those of *Espeletia aristeguietana*, whereas its synflorescence has the branching patterns and shape of *Libanothamnus neriifolius*. The same pattern can be seen when hybridization occurs between the lower-elevation *Espeletia brassicoidea* and the higher-elevation *Espeletia conglomerata*, or with other hybrids. A possible explanation is that the leaves of the higher-elevation parental species are more adapted to the colder conditions than those of a parental species adapted to warmer conditions, and therefore the expression of this character is selected. In the same way, synflorescences of lower-elevation parental species may be more effective in attracting potential pollinators than those adapted to higher-elevation pollinators. What is clear is that there is a fertile field of research on hybridization within the subtribe.

Based on my personal observations and collections of *Nomen nudum*, collectors should be always suspicious about a hybrid individual when any of the following conditions occurs:

- Isolated individuals with no abundant populations: *Nomen nudum* have no pappus in their cypselae, and seeds are dispersed mainly by gravity; therefore collectors should expect to find several individuals of the same species, all related in some degree, and a few sporadic hybrids of sympatric or parapatric species.

- Generally the hybrids have leaves with undulate margins, and often unhealthy appearance.

- Pubescence of hybrids frequently looks disheveled.

### First report of two species and one genus for Colombia

***Espeletia steyermarkii* Cuatrec. Representative specimens. COLOMBIA: Norte de Santander:** en vía a Chitagá, antes del desvío hacia Cornejo, subpáramo y páramo azonal del Carbón, 2798 m, 7°4.13'N, 72°39.25'W, acaule, altura total 1.7 m (individuos en la población de hasta 2 m), altura hasta la roseta 0.6 m, 5 inflorescencias frescas y 2–3 secas, todas largas y bien desarrolladas, con ramas florales extremadamente largas alternas (aunque individuos en la población las presentan también opuestas), un par de brácteas estériles en sección media del escapo, pubescencia de aspecto dorado-leonado, población muy densa, con todos los individuos en flor, 3 Oct 2009, M.Diazgranados & R.Sánchez 3860 (ANDES!, COL!, HECASA!, MO!, US!); ibidem, 2801 m, 7°4.10'N, 72°39.15'W, acaule, altura total 1.8 m (individuos en la población de hasta 2 m), altura hasta la roseta 0.6 m, 5 inflorescencias frescas con 5–10 capítulos cada una, todas largas y bien desarrolladas, con ramas florales extremadamente largas opuestas (a diferencia de la colección MD 3860), un par de brácteas estériles en sección media del escapo, pubescencia de aspecto dorado-leonado, población muy densa, con todos los individuos en flor, 3 Oct 2009, M.Diazgranados & R.Sánchez 3862 (ANDES!, COL!, HECASA!, MO!, US!; photo: Fig. 1C-E).

***Ruilopezia cardonae* (Cuatrec.) Cuatrec. Representative specimens. COLOMBIA: Norte de Santander:** Municipio Toledo, gran Páramo de Tamá, Parque Nacional Natural Tamá, Cuenca del Río Oirá, al comienzo del Cañón del Oirá, sobre la ladera escarpada en el lado colombiano, 7°23.56'N, 72°22.95'W, 3257 m, población abundante de unos 500 individuos maduros, la mayoría con floración pasada o infértiles y unos pocos con flores frescas, altura total 1.3 m, altura hasta la roseta 0.5 m, altura del caule 0.3 m, inflorescencia terminal copiosa, con 16 ramas florales, flores jóvenes blancas que se tornan rosadas cuando maduras y púrpura en la senescencia, 15 Oct 2009, M.Diazgranados & R.Sánchez 3257 (ANDES!, COL!, HECASA!, MO!, US!; photo: Fig. 1F-I).

**Note.** The species had been reported in the Venezuelan slopes of the gran Páramo de Tama, especially along the basin of the Oirá river. One large population was found on the Colombian side. This is the first record of *Ruilopezia* for Colombia.

### Notes about the geographic distribution

The frailejones are widely distributed and abundant in the high Andean forest and páramos of Colombia, Venezuela and, to a lesser extent, Ecuador, where only one species occurs in the north of the country and in the Sierra de Llanganates (Fig. 2A). The northernmost population (-73.8°W, 11°N) grows in the Sierra Nevada de Santa Marta, at -73.8°N. Even though the author has been told about reports of *Libanothamnus neriifolius* in the state of Sucre in Venezuela (approx. -63.5°W, 10.5°N), which would be the extreme NE of the geographic range, no herbarium specimens from this locality have been seen. The southernmost species is *Espeletia pycnophylla*, which grows in the border region between Colombia and Ecuador and has a separate isolated population far south in the Llanganates National Park (-78.5°W, 1.2°S) (Fig. 2B). Nearly 80% of the species occur between -70.5 and -73°W and 5.5 and 9.0°N, along the Eastern Cordillera in Colombia and the Venezuelan Andes. The frailejones grow in 21 states (64%) of Colombia, six in Ecuador (24%) and 12 in Venezuela (52%).

Most of the species of *Nomen nudum* are restricted to a single topographic páramo complex. Similar to island species, they have lost the ability for long-distance dispersal. Seeds lack pappus (except for the scale-like pappus of *Tamania*) and are dispersed by gravity, with no long-distance dispersers known. Only nine species are shared between Colombian and Venezuela, and a few species occur in multiple páramo localities, including *Libanothamnus neriifolius* (in Venezuela), *Espeletia hartwegiana* (in Colombia) and *Espeletia pycnophylla* (in Colombia and Ecuador) as some of the species with the broadest distributions (Fig. 2B). It is notable that these all have various infraspecific taxa, without counting the autonyms (*Libanothamnus neriifolius* has four varieties, and both *Espeletia hartwegiana* and *Espeletia pycnophylla* have two subspecies and two varieties), which denotes a notable morphological variation.

**Figure 3.**
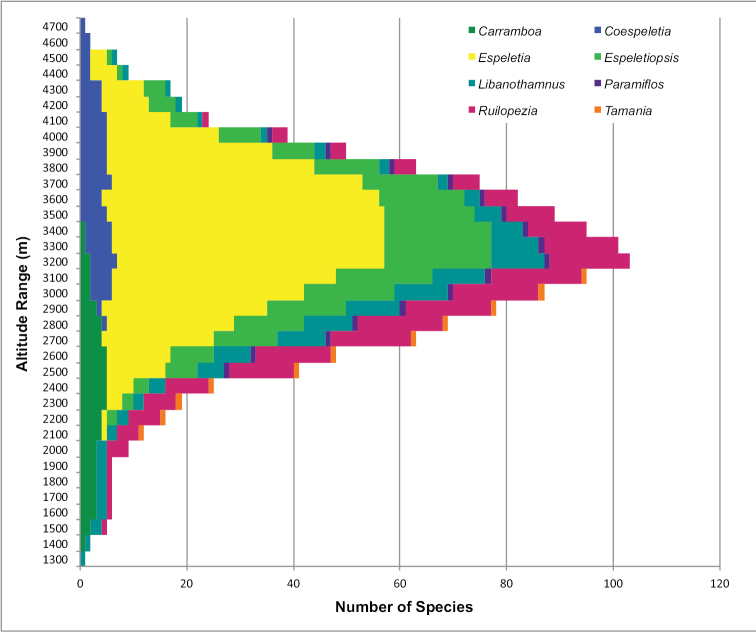
Richness of species throughout the elevation gradient. Each color represents species of each of the genera. *Libanothamnus neriifolius* has been reported as low as 1300 m, while *Coespeletia timotensis* has been found at 4780 m. Most of the species (104) grow in elevations between 3200 and 3400 m.

The frailejones grow abundantly above the tree line of the tropical Andes. Although there are some species endemic from the high Andean forests, and a few that can succeed at elevations as low as 1300 m (i.e. *Libanothamnus neriifolius* var. *turmalensis*, coll. J.Steyermark 105028), most of the taxa (104 species) are found between 3200–3400 m of elevation (Fig. 2C, 3). A few species grow in the superpáramo, close to the snow line, and the highest elevation ever reported is for a *Coespeletia timotensis* (coll. L.Ruiz-Terán 851) growing at 4780 m (Appendix 1). In general, *Carramboa* and *Tamania* grow below 3500 m of elevation, *Coespeletia* grows above 2800 m while the remaining genera have a more or less normal distribution throughout the entire range between 2000–4600 m (Fig. 3, Appendix 1).

There are three apparent centers of radiation: Mérida (with 44 spp.) in Venezuela, Santander–Norte de Santander (39 spp. combined) and Boyacá (45 spp.) in Colombia (Fig. 2E–F). An analysis of richness by area (squares of 0.4° × 0.4° km^2^) highlights two areas with the highest number of species (> 22 spp.), one in central Mérida and the other in Boyacá with limits of Santander (Fig. 2D). Táchira, a Venezuelan state bordering with Colombia, has the highest richness of genera, followed by Mérida (Fig. 2E). Overall, Colombia has the highest richness of species (86 spp.), followed by Venezuela (67 spp.) and Ecuador (1 spp.). These numbers include one presumable species in Colombia (i.e. *Espeletia tillettii*), found on the Sierra de Perijá in the Venezuelan border with Colombia.

The author is currently describing 17 new taxa of frailejones. The phylogeny will provide new insights into understanding the relationships between species and genera, and will generate further changes in the taxonomy of the group. The systematic list here provides a taxonomic base for all these changes and for any other study related to frailejones.

## Appendix

Geographic distribution of the species of the subtribe Espeletiinae. (doi: 10.3897/zookeys.16.3186.app) File format: MS Excell spreadsheet (xlsx).

**Explanation note:** Data based on the evaluation of 3794 herbarium specimens.
